# ZACP: enhancing skin lesion classification ability using zero attention and complete perception

**DOI:** 10.1186/s42492-026-00223-3

**Published:** 2026-07-06

**Authors:** Hailong Zuo, Zhi Weng, Zhenshuai Fu, Fangfang Ding, Jun Chai, Liru Chen, Caili Gong, Zhiqiang Zheng

**Affiliations:** 1 College of Electronic Information Engineering, Inner Mongolia University, Hohhot, Inner Mongolia 010021 China; 2 College of Life Sciences, Inner Mongolia University, Hohhot, Inner Mongolia 010021 China; 3 Imaging Medicine Department, Inner Mongolia AeroSpace Hospital, Hohhot, Inner Mongolia 737399 China; 4 Otolaryngology Department, Xinyi City People’s Hospital, Xuzhou, Jiangsu 221400 China

**Keywords:** Zero attention mechanism, Vision transformer, EfficientNet, Skin lesion, Complete perception

## Abstract

With the increasing number of dermatology patients owing to ecological deterioration and unhealthy lifestyles, hospitals are experiencing significant diagnostic pressure, and patients are suffering from delayed treatment and poor medical experience. Therefore, there is an urgent need to develop accurate skin lesion classification tools. To address this issue, this study proposed the zero attention and complete perception (ZACP), a novel skin lesion classification system based on EfficientNet (EN). ZACP integrates two key modules: ZA and CP. ZA is an improved multi-head attention mechanism that injects zero vectors into key/value vectors, introduces a benchmark to suppress invalid attention to non-semantic regions (e.g., normal skin texture), and enhances the focus on lesions. Derived from the squeeze and excitation (SE) module, the CP embeds the ZA to achieve multiscale attention-retaining SE channel-weighting ability while enabling the ZA to capture local subtle features (e.g., melanoma boundaries) via patch segmentation and positional encoding. Additionally, the original activation function of EN was replaced with Mish to stabilize the training. Evaluated on the authoritative HAM10000 and ISIC2017 datasets, ZACP outperformed the comparative models in terms of accuracy (ACC) and other metrics, achieving 95% and 91% ACC on the two datasets, respectively. The source code is available at: http://github.com/ZACP-DERMATOLOGY.

## Introduction

In contemporary society, harmful substances from industrial emissions, urban air and water pollution pose threats to human skin health. The adverse effects of environmental factors on the skin are becoming increasingly severe [1]. In addition, unhealthy lifestyle habits increase the risk of skin diseases [2]. These include excessive cleansing, chronic sleep deprivation, prolonged use of electronic devices, and a lack of sun protection [3]. These factors interact with each other, making skin health problems more prominent. Due to busy daily work or inefficient medical treatment, people tend to neglect skin diseases, thus missing the optimal treatment stage. Eventually, skin diseases progress to cancer [4].

The traditional method for diagnosing skin diseases is visual examination. However, not all dermatologists have extensive clinical experience, and visual inspection is subjective. Diagnostic differences exist among dermatologists even for the same disease [5]. Moreover, skin diseases exhibit disease heterogeneity, that is, the same disease exhibit different symptoms among different individuals. These differences can be reflected in etiology, pathogenesis, clinical manifestations, disease progression, treatment effects, and prognosis, which pose a considerable challenge to traditional visual inspection [6]. Therefore, when visual inspection cannot meet the diagnostic requirements, most dermatologists resort to tissue biopsies. Although this test has a high accuracy (ACC) rate, patients must visit the hospital in person, queue up for registration, and wait approximately 3–14 days for the diagnostic results. This method is not only time-consuming, but also diagnostically inefficient.

Deep learning has demonstrated an excellent performance in image understanding [7]. Dermoscopic images provide high-quality original lesion image materials for deep learning models, which strongly support the ability of the models to understand images [8]. However, there are several challenges to using deep learning to study skin lesions, as described below.

**Fig. 1:**
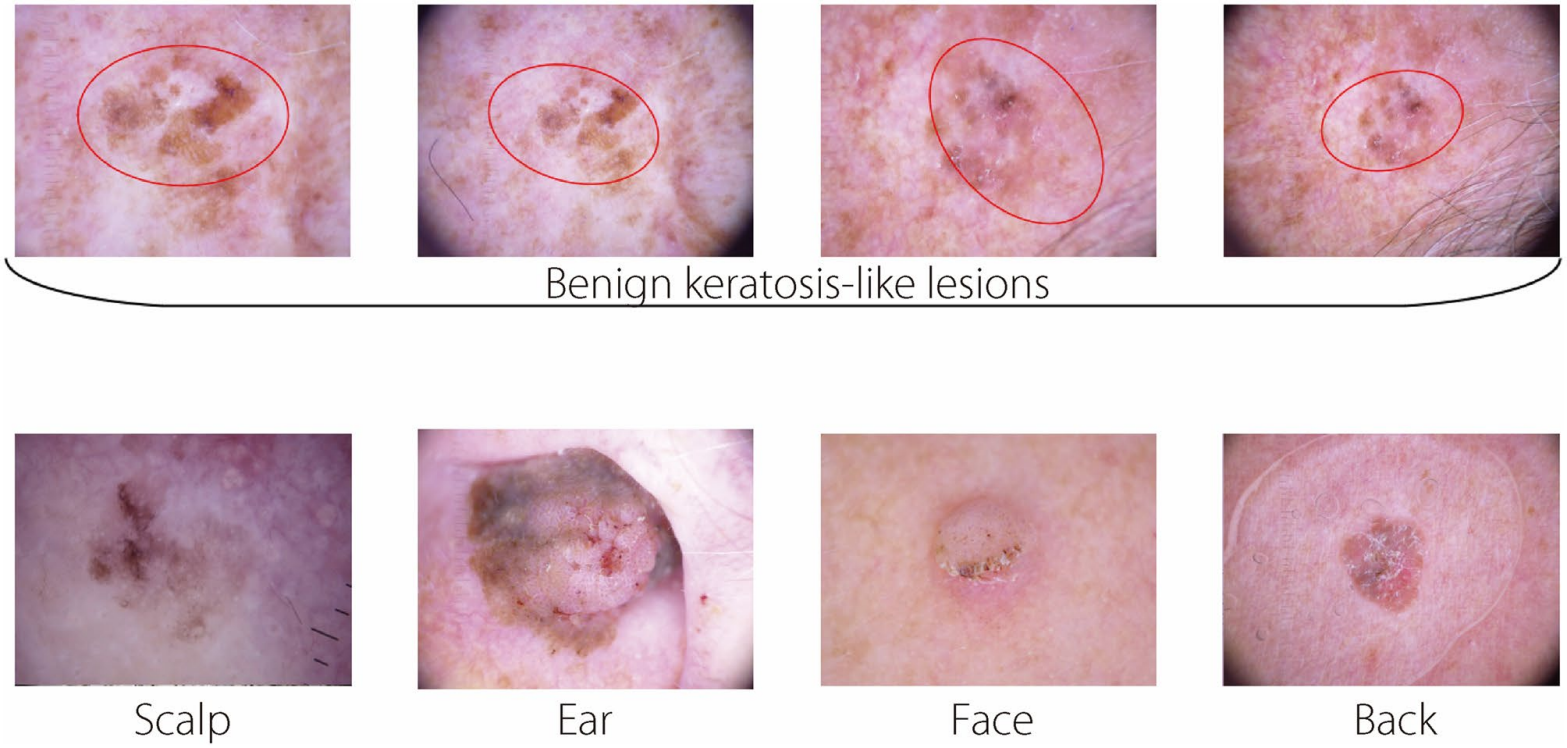
Image differences in the dataset

1. Skin diseases exhibit disease heterogeneity, resulting in diverse manifestations of the same disease, as shown in the first row of images in Fig. 1.
2. When acquiring dermatological images, image quality may be affected by factors such as shooting conditions and device differences. The skin color of patients also has a significant impact on disease diagnosis, as shown in the second row of images in Fig. 1.
3. There is an imbalance between the dataset categories. The number of different types of skin diseases is extremely uneven, and the different characteristics of skin diseases at different stages further exacerbate the problem of category imbalance. For example, in the HAM10000 [9] dataset, there were 6705 melanocytic nevi (NVs) and 142 vascular lesions (VASCs).

Although many image classification methods have been proposed in the field of deep learning [10], there remains room for improvement. Therefore, in this study, we verified the effectiveness of the proposed method on two widely recognized and commonly used datasets: HAM10000 and ISIC2017[11]. We attempted to retain the hierarchical structure of the EfficientNet (EN) [12] network. Thus, we introduced the zero attention and complete perception (ZACP) modules and replaced the activation function in the EN network to improve the ACC of classification results. The contributions of this study are as follows.

- To address the data-imbalance problem in the dataset, we introduced two data augmentation techniques: intraclass augmentation and the proposed multisource image augmentation technique. These techniques can alleviate problems caused by data imbalance and enhance data diversity and quantity.
- We added two additional structures to the ZACP to improve the ACC of the classification system: zero attention (ZA) and complete perception (CP) structures. ZA compresses irrelevant information and highlights important information, and CP strengthens the ability of the model to extract deep visual features, thereby enhancing its ACC.
- We replaced the EN network activation function with a novel Mish activation function. This function can better facilitate the model in improving classification ACC and, to a certain extent, enhance training stability.
- The proposed method demonstrated performance improvements of varying degrees on two datasets: HAM10000 and ISIC2017.

The remainder of this paper is organized as follows. Introduction section reviews the literature on the development of image classification. Methods section elaborates on the technical implementation details and framework of the proposed method, and introduces the datasets, evaluation metrics, and experimental settings. Results section reports and analyzes the experimental results and compares them with those of previous studies. Discussion section presents a discussion of this study. Finally, Conclusions section summarizes the study and suggests future research directions.

### General image classification

#### Convolution from shallow to deep

Image-classification technology in deep learning can be traced back to LeNet [13]. This was the first neural network model to apply convolution to image classification. With the success of the LeNet model, a series of excellent models with traditional convolution modules as the basic structure subsequently emerged, such as GoogleNet [14] and ResNet [15]. These models feature larger depth and width than the initial LeNet; therefore, the hardware requirements are also higher.

To improve the performance of these convolutional networks under limited hardware conditions, an inception module was proposed in GoogleNet. It processes different-sized convolutional kernels in parallel, extracts image features from multiple scales, and enhances the model’s ability to extract features from complex images. Remarkable results were achieved in the image classification tasks. To address the problems of gradient vanishing and explosion, researchers first proposed a residual connection in the ResNet network. This enables neural network models that originally suffer from gradient-related issues to achieve greater depth and dimensionality. Tan and Le [16] conducted in-depth thinking on the width of convolution and image resolution and proposed the EN network for conventional image classification. Compared with neural networks of the same period, it has significantly improved ACC without a substantial increase in the number of parameters, thereby achieving good classification performance. Subsequently, EfficientDet, another EN-based network, adopted the same core compound scaling philosophy and achieved remarkable performance on object detection tasks.

#### Introduction of attention mechanism

While interning at Yoshua Bengio’s laboratory, Bahdanau et al. [17] proposed the concept of a weighted average to address the information bottleneck between the recurrent neural network encoder and decoder in machine translation. Subsequently, at Yoshua Bengio’s suggestion, the concept was renamed ‘attention.’

Researchers in the classification field have noted the important role of attention mechanisms [18]. Therefore, an attention mechanism was introduced into image classification. Hu et al. [19] proposed a squeeze and excitation (SE) structure for measuring the importance of each channel. Zhao et al. [20] introduced visual attention into a classification model to add an attention mechanism similar to that of the human eye to the convolutional neural network (CNN), enabling better image classification. Shang et al. [21] used a pyramid attention mechanism to remove redundant information and enhance the classification network performance. Overall, the introduction of these attention mechanisms improved the classification ACC of general images.

#### Rise of transformer

Through the collaborative efforts of several scientists at Google, a model that relies solely on the attention mechanism was developed. Subsequently, a series of image-processing networks based on transformers emerged [22]. The vision transformer (ViT), proposed in Dosovitskiy et al. [23], is the first transformer-based model for image recognition. It divides an image into multiple patches and introduces these patches in sequence into the transformer, thereby validating the feasibility of applying a transformer to an image domain. Liu et al. [24] present a hierarchical visual transformer model built on a transformer architecture. The adoption of hierarchical feature maps provides a robust backbone for tasks such as object detection and classification. Following the development of ViT, Touvron et al. [25] also used a transformer to develop a new model, DeiT, which achieved a top-1 ACC rate of 83.1% on the ImageNet dataset.

Wang et al. [26] reviewed PVT, a pure transformer backbone that integrates CNN-like local feature advantages with transformer’s global modeling capability. It replaces traditional CNN backbones and serves as a unified backbone for diverse visual tasks without convolutional operations, demonstrating the formidable capabilities of the multihead attention mechanism. The outstanding performance of these variant networks demonstrate the formidable capabilities of the multihead attention mechanism in transformers [27].

### Dermatological image classification

Medical image classification is based on general image classification. Consequently, numerous techniques based on general image classification can be directly applied to medical image classification. Qing et al. [28] proposed a visual feature-flow transformer for classification tasks. This network features a simple structure and achieves high classification ACC. Xie et al. [29] introduced a CNN integrated with an attention mechanism that remarkably enhanced network performance.

However, dermatological image classification significantly differs from traditional object classification methods. Classification of dermatological images requires precise differentiation between various skin diseases. This requires meticulous subdivision of different stages and severity levels of the same skin disease. With more detailed and specialized classification objectives, there is a higher demand for classification ACC and precision. Aboulmira et al. [30] proposed the concept of combining wavelet decomposition with an EN network for dermatological image classification. Wang et al. [31] considered the changes in the appearance of skin lesions and the interference of dermoscopic imaging noise, and proposed a multilevel attentive skin lesion learning network to enhance the classification of melanoma (MEL). Promising results were obtained using the ISIC2017 dataset. Zhao et al. [32] proposed a progressive multistage attention network, enabling the model to gradually shift its focus from stable fine-grained regions to coarse-grained regions, thereby improving the ACC of disease classification [33].

## Methods

### Data augmentation

To address the issue of data-class imbalance in the dataset, we employed enhancement methods that included intraclass enhancement [34] and multisource image region replacement. Data enhancement methods can have varying degrees of positive impact depending on different tasks and datasets. When selecting these enhancement methods, it is essential to consider their potential consequences carefully. The data enhancement method for multisource image region replacement is based on remote sensing. During the processing of remote sensing images, infrared and visible light images are normally combined to achieve data enhancement, thereby improving the performance of relevant models.

**Fig. 2:**
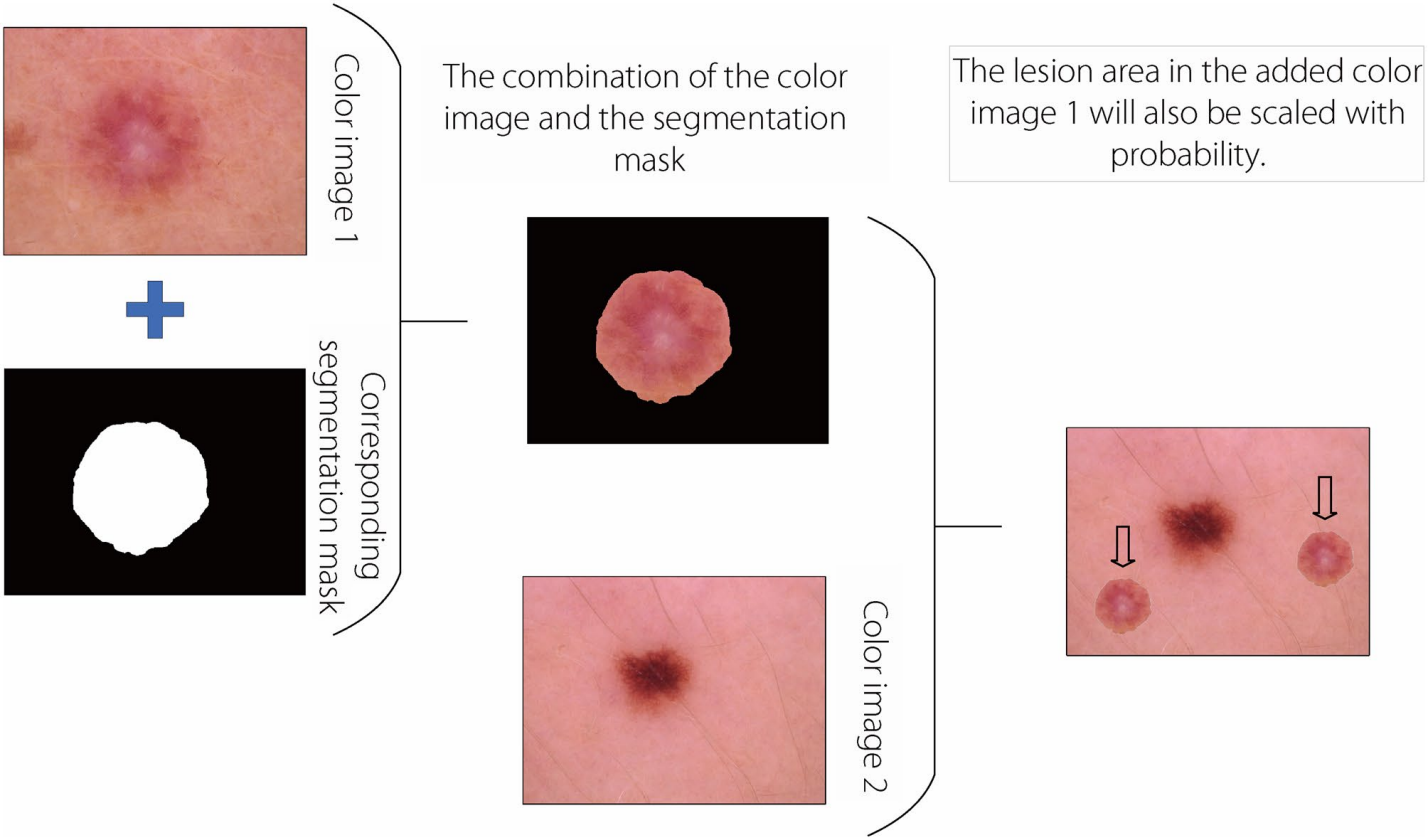
Multisource image fusion method

In this study, we introduced a data enhancement method based on multisource image region replacement and achieved remarkable results. The specific operation process is as follows. For an image with lesions, we first determined the mask area (i.e., marked lesion area). We then randomly selected a non-masked area of an image from another class and added the masked area of the image with lesions to this non-masked area. The number of additions was random and could be one or two. Through such operations, a new image showing the characteristics of two different diseases can be generated. The effect of the newly generated image is shown in Fig. 2.

We employed various specific data transformation methods to implement an intraclass enhancement strategy. First, a rotational operation was performed. We simulated the visual appearance of lesions in different orientations by rotating dermatological images around their geometric centers at different angles. Central region scaling involves magnifying or reducing the central lesion area of the image, which is used to reflect the characteristic differences in lesions at different observation scales. Second, to adjust the color and visual attributes of the images, we changed their brightness and contrast by adjusting their luminance and contrast levels. This generated samples with different visual effects, thereby enhancing the robustness of the model to changes in image brightness and contrast. In addition, the operation of converting a color image into a grayscale image removes the interference of color information, enabling the model to focus more on structural information, such as textures and contours in the image. This structural information is crucial to recognize skin diseases.

The abovementioned intraclass enhancement processing methods were performed with a probability of 50%, which could significantly improve the diversity of the minority classes in the dataset. Increasing the number of samples of these minority classes enables the model to achieve a more balanced learning and understanding of the characteristics of various diseases during the training process, avoiding model bias caused by data imbalance. This plays a key role in the ACC and reliability of medical image classification.

### Backbone network of EN

The backbone architecture of our model was based on EN, which is an image classification model first published at the International Conference on Machine Learning in 2019. Its core innovation is based on a novel model scaling method. Evenly scaling the depth, width, and resolution of the network not only improves the ACC of the model but also reduces the consumption of computational resources. Compared with traditional CNN architectures, EN can achieve better performance while significantly reducing the number of parameters and the amount of computation.

EN adopts various efficient convolution methods in convolutional operations. For example, it uses depthwise separable convolution. This convolution method decomposes a standard convolution into two steps: depthwise and pointwise convolution. Depthwise convolution performs a convolution operation on each input channel separately, substantially reducing the amount of computation and the number of parameters. This significantly reduces the computational cost without compromising the performance. In addition, EN introduces a bottleneck structure using the SE module. In the bottleneck structure, the dimensions of the input are first reduced through a 1 × 1 convolutional layer, reducing the number of channels and thus lowering the computational cost. Subsequently, depth-wise separable convolution and SE operations were performed to extract the features. Finally, a 1 × 1 convolutional layer restores the channels to their original sizes. This structure effectively reduces the amount of computation and the number of parameters while maintaining good feature extraction capabilities. EN is composed of a structure with repeatedly stacked Mobile Inverted Bottleneck Convolution (MBConv) and forms eight different versions, from B0 to B7. The structure of B0 is presented in Table 1 [16].

**Table 1:** Structure composition of B0 version of EfficientNet

Stage *i*	Operator F_*i*_	Resolution H_*i*_ × W_*i*_	Channel C_*i*_	Layer L_*i*_
1	Conv3 × 3	224 × 224	32	1
2	MBConv1, k3	112 × 112	16	1
3	MBConv6, k3	112 × 112	24	2
4	MBConv6, k5	56 × 56	40	2
5	MBConv6, k3	28 × 28	80	3
6	MBConv6, k5	14 × 14	112	3
7	MBConv6, k5	14 × 14	192	4
8	MBConv6, k3	7 × 7	320	1
9	Conv1 × 1 & Pooling & FC	7 × 7	1280	1

*i* denotes the sequential index of the network’s building stage, F_*i*_ represents the feature extraction operator adopted in the *i*-th stage, H_*i*_ × W_*i*_ indicates the spatial resolution (height × width) of the output feature map generated by the *i*-th stage, C_*i*_ refers to the number of output channels of the *i*-th stage, and L_*i*_ stands for the number of repeated convolutional blocks stacked within the *i*-th stage. MBConv: Mobile Inverted Bottleneck Convolution

### Proposed system architecture

**Fig. 3:**
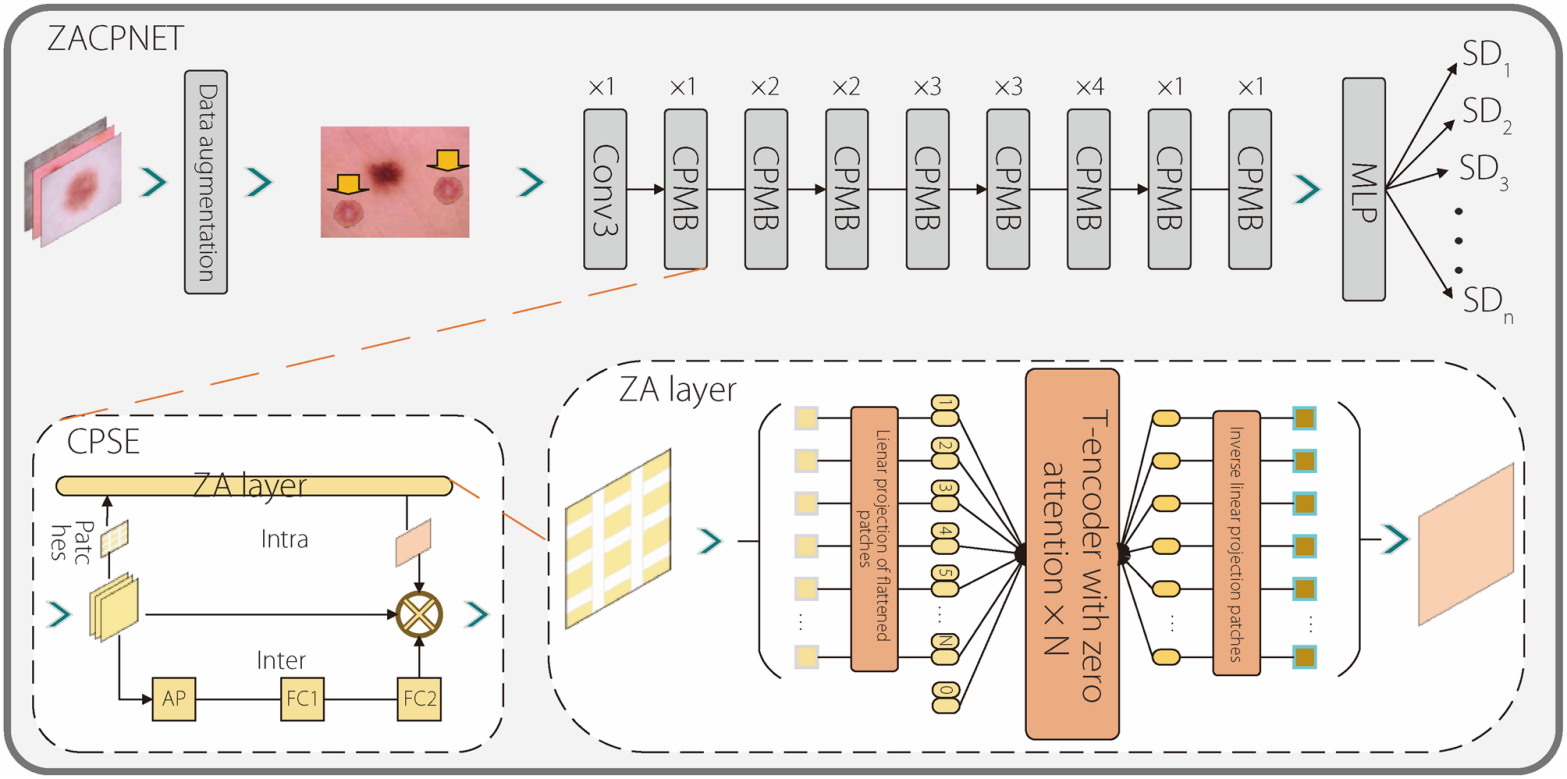
Overall architecture of zero-attention and complete perception. ZACP: Zero attention and complete perception; CPSE: Complete perception squeeze and excitation; ZA: Zero attention; CPMB: Complete perception and mobile inverted bottleneck convolution

The ZACP architecture consists of three main parts: image enhancement, EN backbone, and CP and mobile inverted convolution. As mentioned earlier, before the input dermatological images are entered into the model, they undergo preprocessing. This step is important because of class imbalance in dermatological disease datasets, and preprocessing can effectively alleviate this issue, providing a solid foundation for the accurate training of subsequent models. Only after the images are carefully processed that they are used as inputs for the proposed model. This process is shown in Fig. 3.

The enhanced images were processed using the proposed ZACP network. The network structure of the ZACP comprises nine stages. Notably, the number of layers in each stage follows the original EN network structure. Thus, it not only exploits the advantages of the well-established EN network architecture but also provides a stable basic framework for subsequent improvements. A classifier was set up in the final stage of the ZACP network. It predicts the final class probability of an image according to the features extracted in the previous stages to determine the type of skin disease. In this study, we were fully aware of the advantages of the EN structure; therefore, we retained its overall framework as the basis. To further improve the performance of the model, we optimized its key components. Specifically, we replaced the original MBConv structure with a complete perception and mobile inverted bottleneck convolution (CPMB) structure. The MBConv structure plays an important role in the traditional EN network. This strengthens the network’s ability to extract visual features through stacking, as presented in Table 1. However, in our in-depth study, we found that the MBConv structure has certain limitations. The CPMB structure innovatively integrates transformer technology. This ingenious design enables the model to take advantage of the powerful attention mechanism of the transformer to focus more accurately on key areas of the image during image processing. Consequently, it significantly improves the capture and analysis of key information in lesion images, thereby providing stronger support for the accurate diagnosis of skin diseases.

#### CPMB module

As a cross-channel attention mechanism, the SE module in the MBConv structure enhances the network’s ability to focus on and filter information from different channels to a certain extent. However, this module has several limitations. It only considers the relationships between channels, while ignoring the semantic correlations within a single-channel region. This makes the model unable to fully consider the importance of different regions within each single channel, causing some crucial local information to be overlooked during processing.

**Fig. 4:**
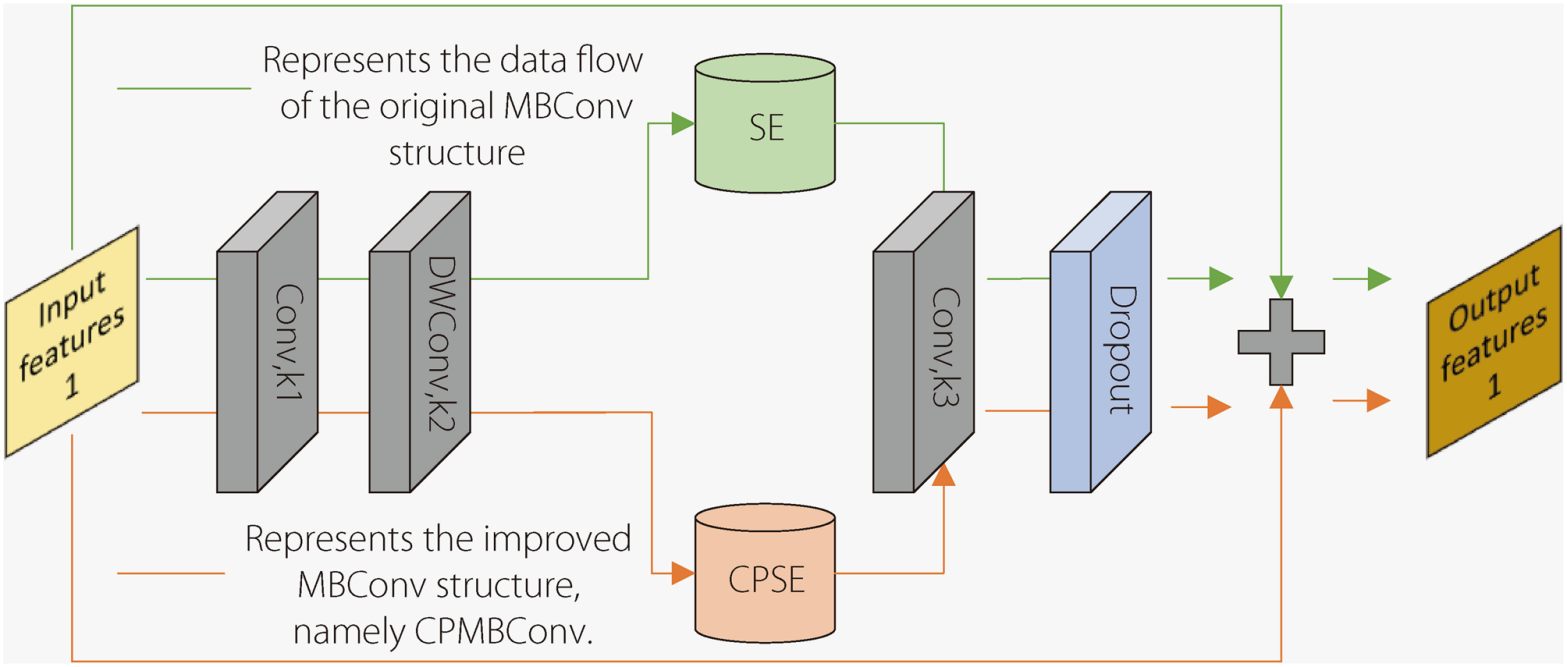
Overview of the complete perception and mobile inverted bottleneck convolution structure

Thus, we made targeted improvements to the SE module, the improved structure of which is shown in Fig. 4. The green lines in the upper part indicate the data flow direction of the original MBconv structure, whereas the orange lines in the lower part indicate the data flow diagram after improvement. The figure clearly shows that this improvement is mainly caused by replacing the SE structure with the complete perception squeeze and excitation (CPSE) structure. Through this replacement, the new CPSE structure can not only focus on the attention weight information of different channels, but also capture the semantic information within a single channel more comprehensively. This enhances the ability of the model to perceive each region in the image, thus providing richer and more accurate feature information for subsequent image analysis tasks.

#### CPSE module

The detailed structure of the CPSE module is shown in the lower-left part of Fig. 3. This module is composed of two key parts: the ZA and SE. In this architecture, the SE component continues its function in the traditional structure, which is to handle the attention mechanism between channels. By evaluating and weighting the importance of different channels, the model can focus more effectively on the information from different channels, thereby enhancing its ability to grasp the overall features. The ZA component focuses on the attention mechanisms of the different regions within a single channel. This innovative design enables the model to focus further on key regions within the channel, thereby improving its ability to perceive and analyze local details.

The detailed structure of the ZA layer is shown in the lower-right portion of Fig. 3. The workflow was relatively elaborate and complex. First, a segmentation operation was performed on the convolutional features. The segmentation granularity is a hyperparameter that has an important impact on the model performance: the more patches are divided, the more detailed the local information captured by the model, and theoretically, the better the classification ACC. After segmentation was completed, a linear mapping operation was used to convert these segmented patches into a series of patch vectors that carry the feature information of the local regions of the image. Position encoding was added to enable the model to better understand the position information of these patch vectors in the image. It should be noted that the dimension of position encoding is also a hyperparameter, and its reasonable setting is crucial for the model to capture spatial information accurately. Different dimensional settings may have significant effects on the performance of the model. This formula is expressed as follows:

1 $$F = {R^{C \times H \times W}}$$

2 $$N = {{H \times W} \over {h \times w}}$$

3 $${V_i} = L\left( {{P_i}} \right),i = 1,2,3 \ldots$$

4 $${u_i} = concat\left( {{v_i},{e_i}} \right),i = 1,2,3, \ldots$$

where *F* denotes the convolutional feature, *N* is the number of patches with the same segmentation size, *V*_*i*_ is the vector of patches after linear convolution, and *u*_*i*_ is the vector of patches with the positional encoding *e*_*i*_ added.

#### ZA in multihead attention

**Fig. 5:**
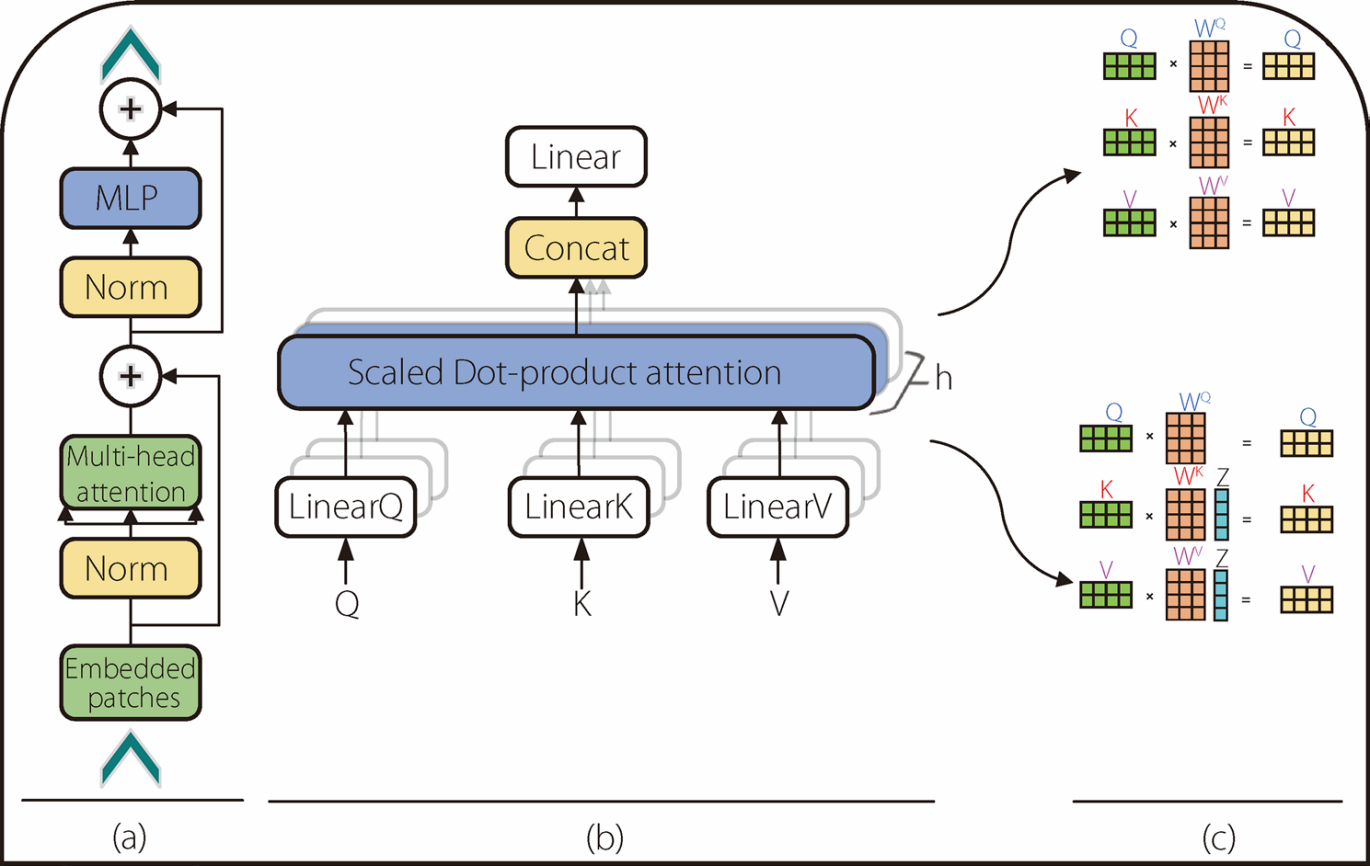
Comparison diagram of the calculation processes between the original multihead attention and improved multihead attention

In the calculation process of the traditional multihead attention mechanism, the query vector *q* and key vector *k* are multiplied to calculate the scores, as shown in Fig. 5(b). This type of scoring is essentially relative. For instance, assuming that there are three query vectors *q*1, *q*2, and *q*3, when calculating the attention score of *q*1, the dot products of *q*1 with each key vector are computed. If these three query vectors do not contain important semantic information within the current task scenario, the calculated scores are typically low. However, after processing using the softmax function, these scores are transformed into attention weights in the form of a probability distribution. Although all three scores are low, the attention weight corresponding to the highest score still accounts for a substantial proportion of the entire probability distribution, thus receiving a larger attention allocation. This situation leads to the emergence of attention bias, causing inaccuracies when the model focuses on information. Consequently, this will have a negative impact on the subsequent extraction of key information and interfere with the model’s accurate comprehension and analysis of important content, as illustrated in Fig. 5.

Traditionally, most improvement strategies for the multihead attention mechanism have focused on adjusting the attention calculation method, adding additional attention modules, and in other directions. However, considering the problems in the traditional multihead attention mechanism mentioned above, we proposed a unique improvement method. We artificially added a column (or row) of zero vectors to the Key (*k*) and Value (*v*) vectors involved in the multihead attention mechanism. The idea of introducing a benchmark by adding zero vectors is unique among the numerous improvement methods. This operation is equivalent to introducing a benchmark reference into the multihead attention mechanism. When attention calculations are performed, the scores can be adjusted according to this benchmark. When encountering the problem of attention bias caused by generally low scores, as described previously, the corresponding scores can be made as low as possible by calculating them using this zero vector. Owing to the existence of “zero attention” after processing using the softmax function, the attention weights corresponding to the vectors with low scores caused by unimportant information become extremely small, and in extreme cases, they may even approach 0. In this manner, the model can allocate attention more reasonably, avoid excessive attention to unimportant information, and thus improve the ACC of key information extraction. The calculation order is shown in sequences in Fig. 5.

$$Q \in {R^{{L_q} \times {D_k}}},K \in {R^{{L_k} \times {D_k}}},V \in {R^{{L_k} \times {D_v}}}$$$$S \in {R^{{L_q} \times {L_k}}}$$ are the query vector, key vector, value vector, and score calculation, respectively. After adding a row (column) of zero vectors, it becomes

5 $${K^\prime } = \left[ {\matrix{ K \cr {{0_1} \times {d_k}} \cr } } \right] \in {R^{\left( {{L_k} + 1} \right) \times {d_k}}}$$

6 $${V^\prime } = \left[ {\matrix{ V \cr {{0_1} \times {d_v}} \cr } } \right] \in {R^{\left( {{L_k} + 1} \right) \times {d_v}}}$$

${K^\prime}$ and ${V^\prime}$ are the matrices of *k* and *v*, respectively, after adding the zero vectors. Here, it represents a zero vector of length *n*.

7 $${S^\prime } = {{Q{{\left( {{K^\prime }} \right)}^T}} \over {\sqrt {{d_k}} }} = \left[ {{{Q{K^T}} \over {\sqrt {{d_k}} }}{{Q{0_1}^T \times {d_k}} \over {\sqrt {{d_k}} }}} \right] = \left[ {S\;{0_{Lq}} \times 1} \right] \in {R^{{L_q} \times \left( {{L_k} + 1} \right)}}$$

Subsequently, the softmax function is applied to the new attention matrix $S^\prime$ to obtain the new attention weight matrix $A^\prime$.

8 $${A^\prime } = soft\max \left( {{S^\prime }} \right)$$

For each row i in $A^\prime$, its elements are calculated as follows:

9 $${A^\prime }_{i,j} = {{\exp \left( {{S^\prime }_{i,j}} \right)} \over {\sum\nolimits_{l = 1}^{{L_k} + 1} {\exp \left( {{S^\prime }_{i,j}} \right)} }},j = 1, \ldots ,{L_k} + 1$$

Equation (9) indicates that when the original *S*_*i,j*_ values are generally low, owing to the newly added score term of 0 after applying the softmax function, the attention weights of the elements with lower scores are significantly reduced. There is no situation in which elements with relatively low scores obtain relatively high weighting scores.

### Datasets

In this study, we used two authoritative and widely adopted datasets: HAM10000 and ISIC2017.

- The HAM10000 dataset, which was a human-to-machine dataset with 10,000 training images, contained 10,015 clinical images of skin lesions. These images were sourced from a wide range of dermatology clinics in Australia, Austria, Spain, and other regions to ensure diversity and representativeness of the data. The dataset encompassed seven common types of skin lesions, including MEL, melanocytic NV, and basal cell carcinoma (BCC), and each image was accompanied by a corresponding disease label. This dataset has been widely used in medical research and machine learning. It has not only enabled researchers to analyze the characteristics and patterns of skin lesions but also served as a common data source for training skin lesion classification models and developing automated diagnosis systems.
- The ISIC2017 dataset was the dataset released by the International Skin Imaging Collaboration in 2017. It was divided into a training set (2000 images), validation set (150 images), and test set (600 images). It focused on three types of skin lesions: MEL, keratinocyte carcinoma, and benign NV. In addition to providing disease classification labels, they provided boundary annotations (segmentation masks) for the lesions. Thus, the ISIC2017 dataset plays an important role in skin lesion segmentation research, skin disease classification, and medical education. They can be used to develop segmentation algorithms, train classification models, and provide rich learning resources for medical learners.

### Evaluation metrics

ACC: ACC describes the degree of closeness between a set of measured and true values. This reflects the reliability of a model for the overall classification task. Specifically, it examines the proportion of samples that the model correctly classified (including correctly identified positive and negative samples) among all samples. The calculation is described in Eq. (10).

10 $$Accuracy = {{TP + TN} \over {TP + TN + FP + FN}}$$

Sensitivity: Sensitivity is used to measure the ability of a model to identify actual positive samples. That is, it represents the proportion of truly positive samples that the model can correctly identify among all positive samples. In many practical scenarios, particularly in the field of medical diagnosis, this indicator is important because it is related to the timely detection of individuals with actual problems. The calculation is described in Eq. (11).

11 $$Sensitivity = {{TP} \over {TP + FN}}$$

Specificity: Specificity reflects the level at which a model accurately identifies actual negative samples. In other words, it is the proportion of samples that the model correctly identifies as negative among all negative samples. In practical applications, high specificity avoids misjudging normal situations as abnormal, reducing unnecessary subsequent operations. The calculation is described by Eq. (12).

12 $$Specificity = {{TP} \over {TN + FP}}$$

Precision: Precision measures the ACC of the prediction results of a model. Specifically, this refers to the proportion of samples that are actually positive among those predicted to be positive by the model. High precision indicates that the positive prediction results obtained by the model are relatively reliable. Its calculation is described by Eq. (13).

13 $$Precision = {{TP} \over {TP + FP}}$$

Weighted average (W.Avg): When dealing with datasets in which the number of samples in each category varies significantly, that is, there is a data imbalance problem, it is particularly important to use the weighted average to calculate metrics such as precision and recall. It assigns weights according to the number or importance of samples in different categories to comprehensively evaluate the performance of the model. The calculation is described by Eq. (14).

14 $$W.Avg = {{{w_1} * {p_1} + {w_2} * {p_2} + \ldots + {w_n} * {p_n}} \over {{w_1} + {w_2} + \ldots + {w_n}}}$$

Macro-average (MAvg) AUC is computed by averaging the AUC values obtained from the one-*vs-*rest ROC curves of all classes, assigning equal weight to each class regardless of its sample size. This metric reflects the model’s overall ability to distinguish between classes while preventing majority classes from dominating the evaluation.

Confusion matrix: The confusion matrix is a crucial tool for evaluating the performance of classification models. It presents the relationship between the prediction results of the model and the actual labels in the form of a matrix. Each column of the confusion matrix represents the predicted class of the model and each row represents the true class of the samples. Each element in the matrix indicates the number of samples for a combination of the predicted and true classes. By analyzing the confusion matrix, we can intuitively understand the performance of the model for each class and identify its strengths and weaknesses.

From the perspective of dermatologists, sensitivity and specificity are particularly important in the diagnosis of skin diseases. This sensitivity index ensures that doctors can detect patients who actually have skin diseases as accurately as possible, thereby reducing the risk of missed diagnoses. The specificity index enables doctors to accurately rule out individuals who do not actually have skin diseases, reducing the probability of misdiagnoses. The combination of these two indices is essential for accurate diagnosis and effective management of patients.

### Experimental setup

In this study, we conducted experiments on the Windows 11 operating system and employed the PaddlePaddle framework. Tests were performed on a system equipped with an NVIDIA Tesla V100 GPU, a 4-core Intel CPU, and 32 GB of system memory. We used the PaddlePaddle 3.0 beta version and trained the model on the HAM10000 and ISIC2017 datasets. To adapt to the different characteristics of the datasets, we set batch sizes of 16 and 8 for these two datasets, respectively. It should be noted that our model was trained for 60 iterations, and the entire process required approximately 8 hours. For the other configurations, we ensured they remained consistent with the settings used during the training of EN and ViT. In addition, we used weights pre-trained on ImageNet to enhance the performance and generalization ability of the model.

**Fig. 6:**
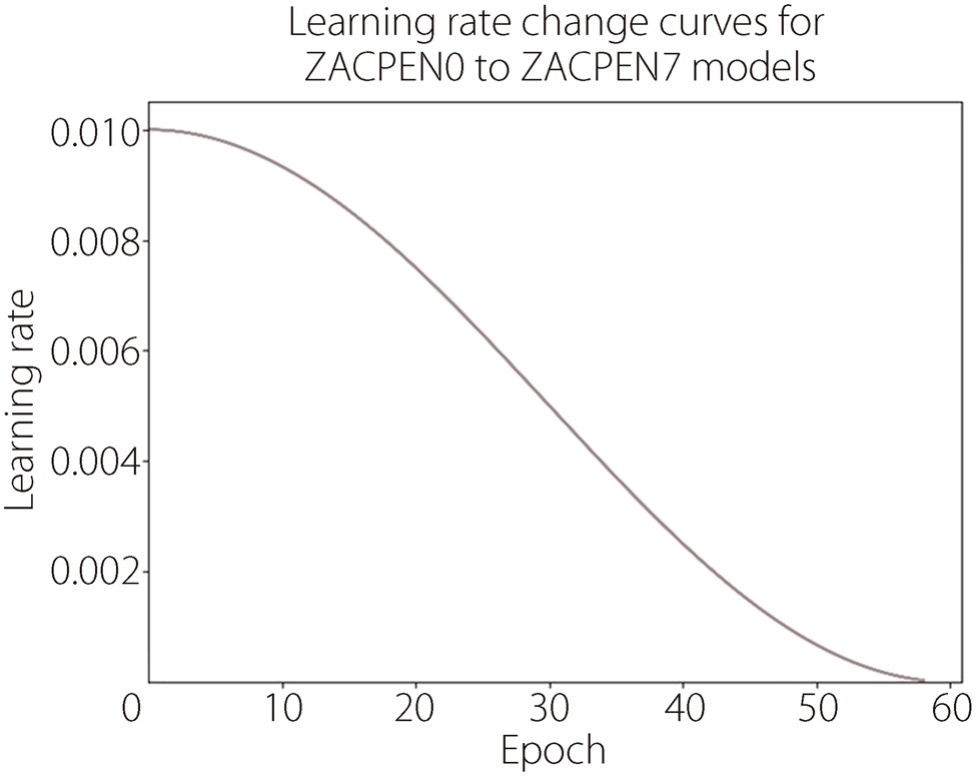
Learning rate curve

Figure 6 shows the learning rate decay curve used in all our test results (ZACPEN0–ZACPEN7). In the initial stage of training, a relatively high learning rate allowed the model parameters to update quickly, advancing rapidly toward the optimal solution and effectively accelerating the convergence speed and reducing the training time. As the training progressed, the learning rate gradually decreased and the update step of the model parameters decreased. This enabled fine tuning when approaching the optimal solution, preventing oscillations near the optimal solution, thereby improving convergence ACC and data fitting.

## Results

### Results on HAM10000 dataset

#### Performance comparison with other benchmark models

To verify the effectiveness of the proposed method, we evaluated the model on the HAM10000 dataset using four metrics: precision, ACC, weighted-average F1-score (W. Ave/F), and macro-average (MAvg) AUC. The results are listed in Table 2.

**Table 2:** Results of the proposed model and other existing models such as VGG16 [35], ResNet-50, MobileNet [36], DenseNet-121 [37], SENet, EfficientNet-B4, and ViT

Method	Precision↑	Accuracy↑	W.Ave/F↑	MAvg AUC↑
VGG16	0.82	0.812	0.897	0.901
ResNet-50	0.86	0.848	0.909	0.925
MobileNet	0.81	0.801	0.915	0.913
DenseNet-121	0.802	0.81	0.917	0.917
SENet	0.854	0.863	0.925	0.939
EfficientNet-B4	0.863	0.876	0.925	0.940
MASLL		0.873		
VIT	0.928	0.947	0.938	0.974
B7 (ours)	**0.931**	**0.959**	**0.94**	**0.987**

The bold values represent the highest scores, and ↑ indicates that the larger the value, the better the performance. W.Ave/F: Weighted-average F1-score; MAvg AUC: Macro-average area under the ROC curve

Table 2 presents a comprehensive comparison of the performance of the proposed model with other models using all the evaluation metrics. Notably, we directly adopted data provided in the original papers. For data not provided in the original papers and models where the exact results were not fully reported, we reproduced their experiments under the identical setup (dataset, network architecture, and hyperparameters) as specified in the original studies. As presented in Table 2, the proposed method achieved the best scores and outperformed the other benchmark models for all the indicators. Compared to the second-best ViT model, the precision, ACC, and weighted average of the proposed model increased by 0.3%, 1.2%, and 0.2%, respectively. Notably, the increase in the MAve AUC score was the largest, reaching 1.3%, further highlighting its excellent performance. The results of the proposed model also prove its superiority and effectiveness compared with other benchmark models.

The results from Table 2 not only confirm the effectiveness of the proposed method compared with other baseline methods, but also demonstrates its excellent performance in the dermoscopic image classification task.

#### Confusion matrix and training variation curve on HAM10000

**Fig. 7:**
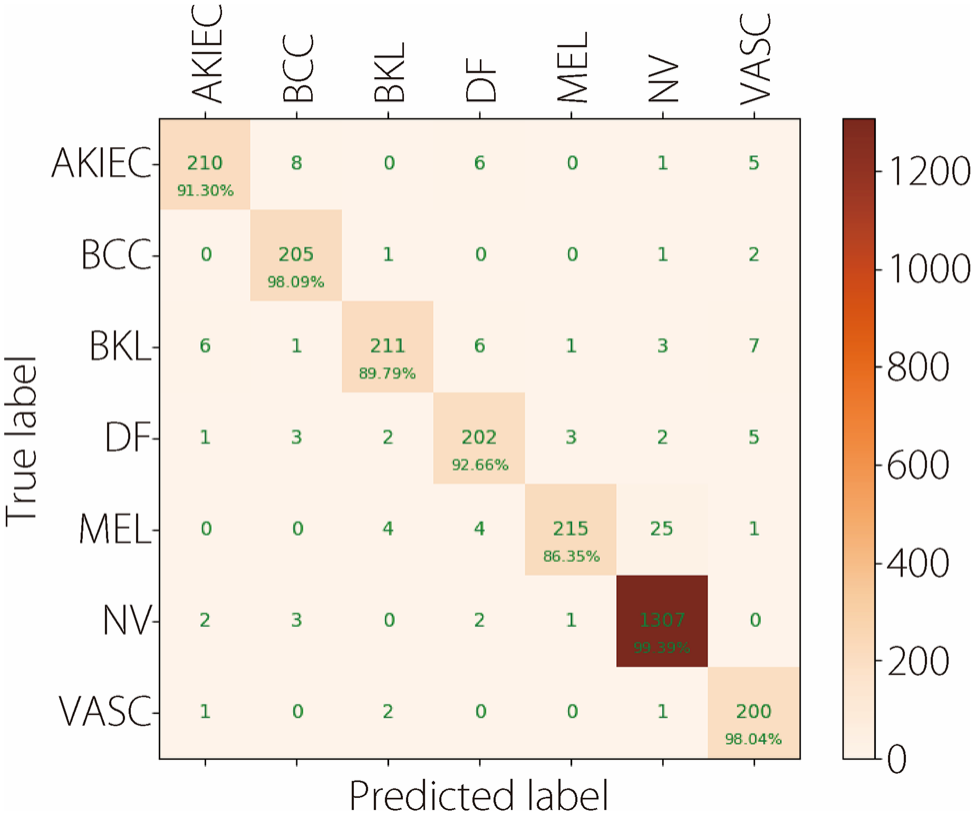
Confusion matrix

Figure 7 shows the best confusion matrix obtained using the improved method. As can be seen from Fig. 7, the multiclass classification model constructed in this study classified seven types of skin lesions: actinic keratosis and intraepithelial carcinoma (AKIEC), BCC, benign keratosis lesion (BKL), dermatofibroma (DF), MEL, NV, and VASC. The results presented in the confusion matrix indicate that the classification performance of the models exhibited different characteristics. Overall, the model exhibited a certain degree of ACC and discriminatory ability. Some categories achieved a high level of classification ACC; however, there is still room for improvement in certain categories.

The classification accuracies for the BCC and VASC categories were 98.09% and 98.04%, respectively. This indicates that the feature extraction and classification decision-making of the model for these two types of samples were relatively accurate, and the model effectively distinguished them from other categories. The classification accuracies for the AKIEC, DF, and NV categories ranged from 91.30% to 92.66%. Although there was a certain degree of misclassification, the overall performance was acceptable. However, the classification accuracies for the BKL and MEL categories were relatively poor with accuracies of 89.79% and 86.35%, respectively. BKL was easily misclassified into AKIEC, DF, and other categories, and there were many misclassifications between MEL, DF, and NV. This indicates that the model may not have fully captured the unique feature information of these categories and that deficiencies existed in the definition of category boundaries.

**Fig. 8:**
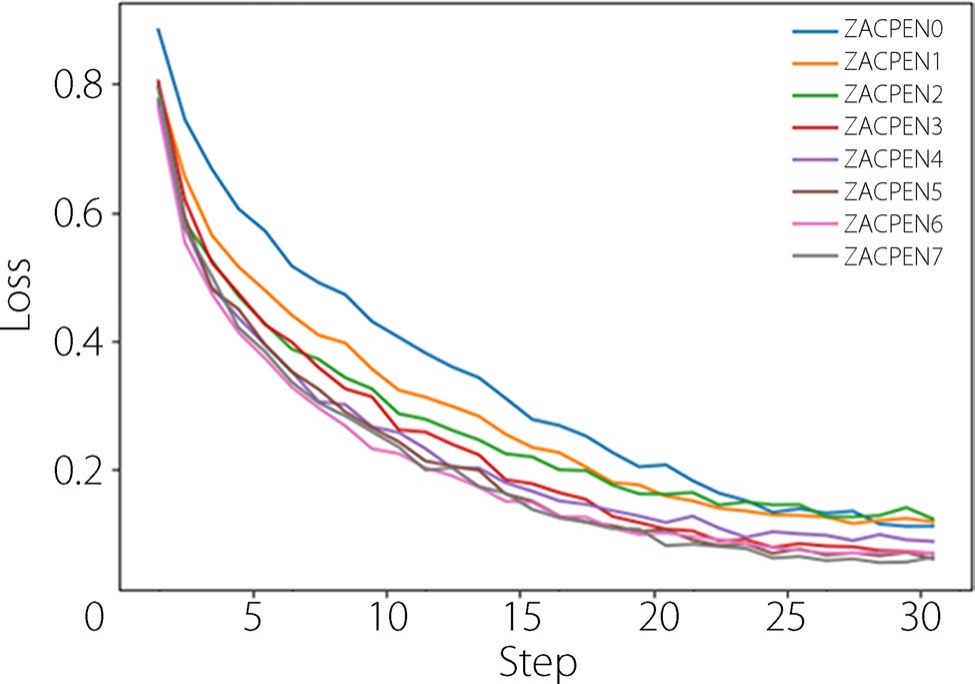
Training loss of ZACPEN0 to ZACPEN7 models

**Fig. 9:**
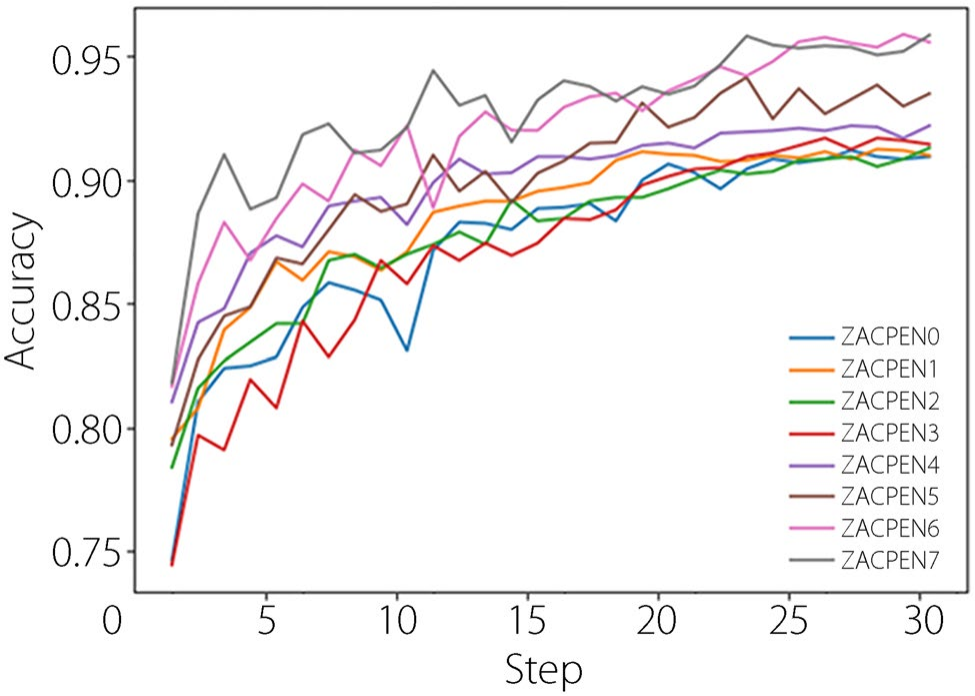
Accuracy of ZACPEN0 to ZACPEN7 models

Figures 8 and 9 illustrate the variations in the training loss and ACC of ZACPEN0 to ZACPEN7 models with respect to the training steps (generating results every two epochs). In Fig. 8, the vertical axis represents the loss value, and the horizontal axis represents the training steps and shows the decreasing trend of the model’s error during the training process. In Fig. 9, the vertical axis represents the ACC, and the horizontal axis represents the training steps and shows the change in the proportion of correct predictions of the model as the training progresses.

As shown in Fig. 8, the training losses of all models were relatively high during the initial stage of training. However, as the number of training steps increased, the loss values decreased rapidly. In the later training stages, the loss values for each model tended to be relatively low and similar. This indicates that the models can effectively learn the data features during training and reduce errors. As shown in Fig. 9, the training ACC of each model was relatively low at the beginning of training and then gradually increased. The ACC of some models reached approximately 95% in the later stages of training, demonstrating that the models possessed good classification capabilities. Although there were slight differences in the ACC improvement rates and final levels among the models, they all exhibited an overall upward trend, indicating that these models were optimized in the correct direction during training.

Overall, both two sets of images indicate that the eight classification models performed well during the training process. The continuous decrease in the loss value and steady increase in ACC suggest that the models could learn effective patterns from the training data. By the end of the training, the models achieved relatively high classification performance and could make relatively accurate classification predictions for the data. This implies that these models exhibit a certain degree of reliability and effectiveness in practical applications.

#### Grad-weighted class activation mapping attention class activation map

**Fig. 10:**
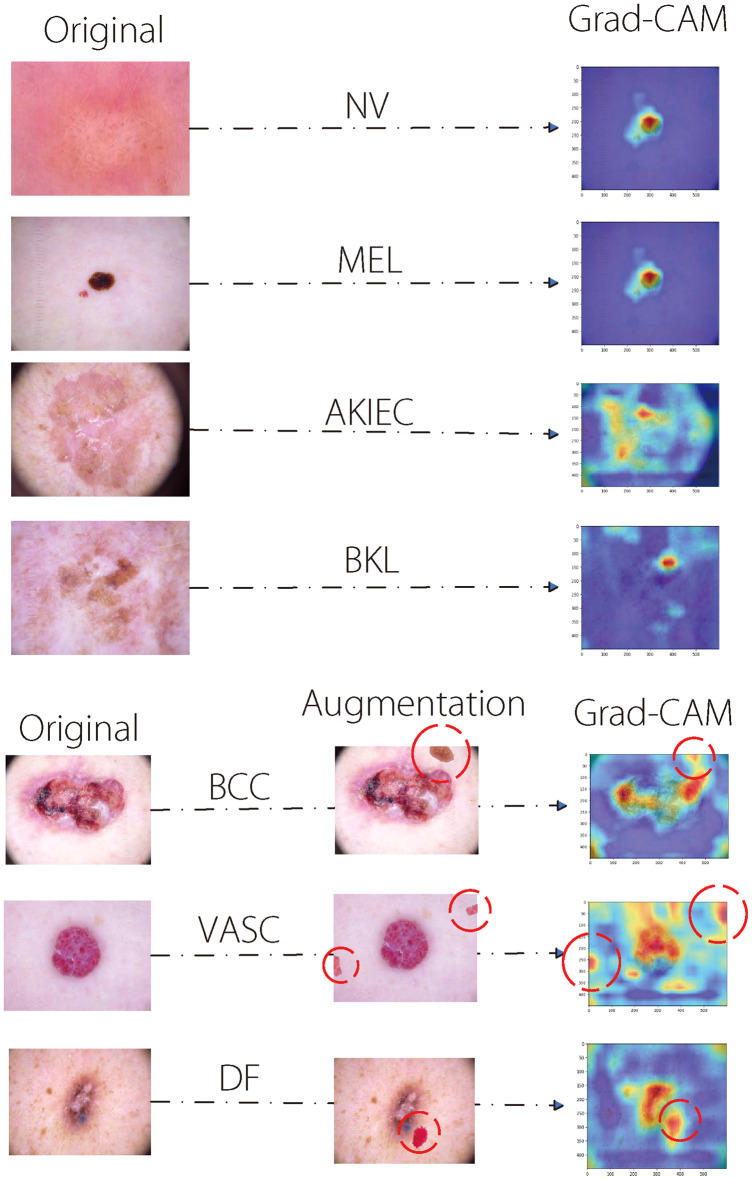
Gradient-weighted class activation mapping on HAM10000 dataset. AKIEC: Actinic keratosis and intraepithelial carcinoma; BCC: Basal cell carcinoma; BKL: Benign keratosis lesion; DF: Dermatofibroma; MEL: Melanoma; NV: Nevus; VASC: Vascular lesion; grad-CAM: Gradient-weighted class activation mapping

Gradient-weighted class activation mapping (grad-CAM) is a visualization technique used to interpret the decision-making mechanism of CNN. It can generate heatmaps to intuitively present the key regions of an image that the model focuses on when making a judgment. This enables researchers to determine whether a model focuses on truly valuable features. Figure 10 shows the grad-CAM visualization results obtained using the HAM10000 dataset.

The left side of the Fig. 10 shows a comparison between the original skin lesion image and the corresponding grad-CAM visualization image. This comparison clearly shows that, with the synergistic effect of the CP module, the proposed method could relatively accurately locate the key regions in the image. For the different types of skin diseases, the attention patterns of the model varied. For MEL and NV diseases, the model must focus only on a small part of the image to accurately determine the type of skin disease. This indicates that these two diseases may have unique and easily identifiable local features. For AKIEC, the model must focus on a relatively large area to make a judgment, suggesting that the features of this type of disease are more widely and diffusely distributed. The situation of BKL was between the above two cases. Although the model primarily focused on a small part of the area, it also considered the remaining part, indicating that its features had relatively prominent key parts and some auxiliary distribution characteristics. The right half of the figure shows the grad-CAM visualization results obtained after enhancing the original image. After embedding images of other diseases in parts of the original image, except the central area, the grad-CAM results show that the model exhibited the characteristics of multipoint attention. Specifically, for each embedded image area of the disease, the model could perform sufficient attention and ensure the correct prediction of the overall image. This phenomenon demonstrates that the image enhancement method adopted in this study exhibits a certain level of effectiveness, which can guide the model to capture more dimensional feature information, thus improving its recognition ability. Experiments were also performed on the ISIC2017 dataset to verify the effectiveness and generalization ability of the enhancement method more comprehensively.

In summary, the experimental results clearly showed that the CP module used in this study played a significant role in guiding the attention distribution of the model. This not only provides solid empirical support for the effectiveness of the proposed method, but also provides an important theoretical foundation for subsequent in-depth research and method optimization. This is expected to achieve more meaningful research results in related fields, such as the intelligent diagnosis of skin diseases.

### Results on ISIC2017 dataset

#### Comparison of the ACC between the ZACP model and the standard EN series models on the ISIC2017 dataset

**Fig. 11:**
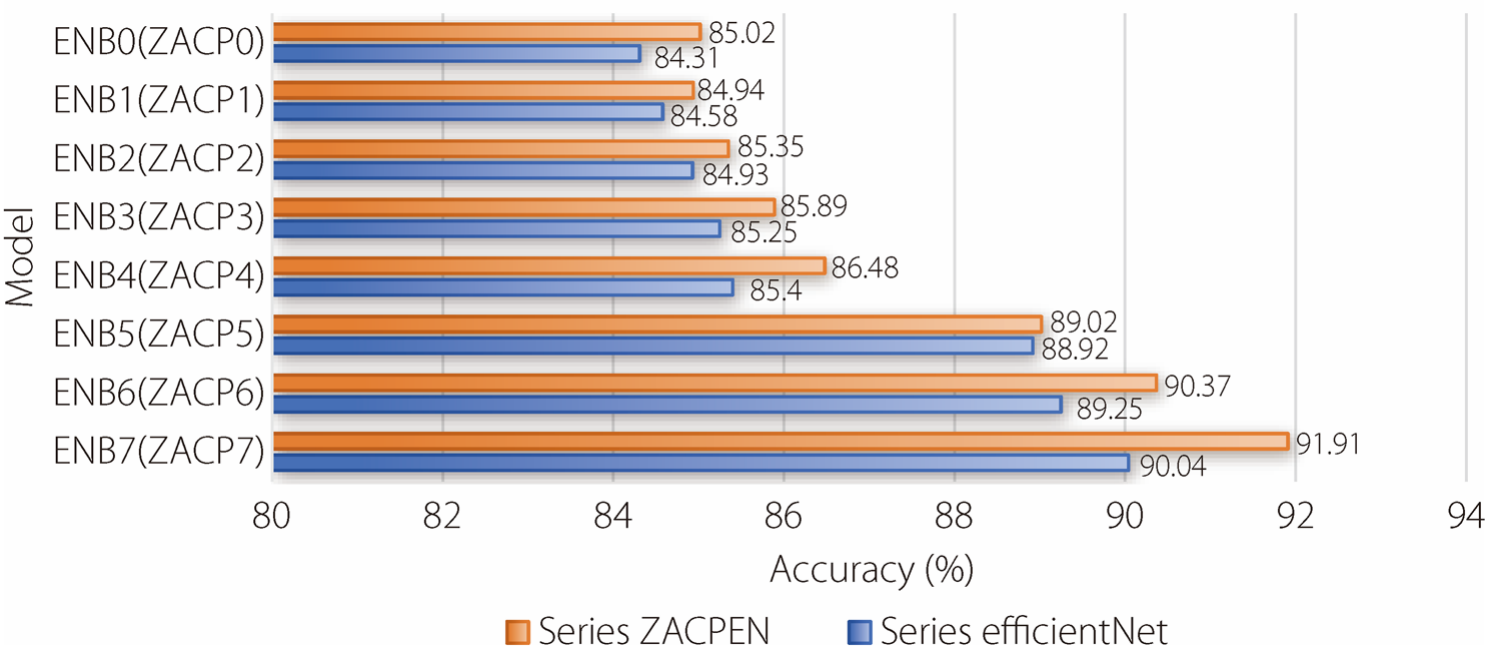
ZACP *vs* EN on the ISIC2017 dataset. ZACP: Zero attention and complete perception; EN: EfficientNet

We conducted experiments using the ISIC2017 dataset for further verification. Figure 11 shows the performance comparison in terms of ACC between the ZACP and the standard EN network on the ISIC2017 dataset.

Based on this comparison figure, the improved models of the ZACP series based on ZA and CP exhibited significant advantages compared with the original EN series models. Comparing each corresponding version, the ACC rates of the ZACP series models almost completely surpassed those of the EN series. Considering the B7 version as an example, the ACC rate of ZACP7 reached 91.91%, whereas that of ENB7 was only 90.04%. In the B6 version, the ACC rate of ZACP6 was 90.37%, which was higher than the 89.25% of ENB6. Even in the B0 version with a relatively small gap, the 85.02% of ZACP0 was higher than the 84.31% of ENB0. This demonstrates that improving the model using the new methods of ZACP, it achieved a stronger classification ability and higher prediction ACC in the three-class classification task performed on the ISIC2017 dataset. This effectively enhanced the model performance, achieving remarkable improvements.

#### Comparison between ZACP and other models on the ISIC2017 dataset

**Fig. 12:**
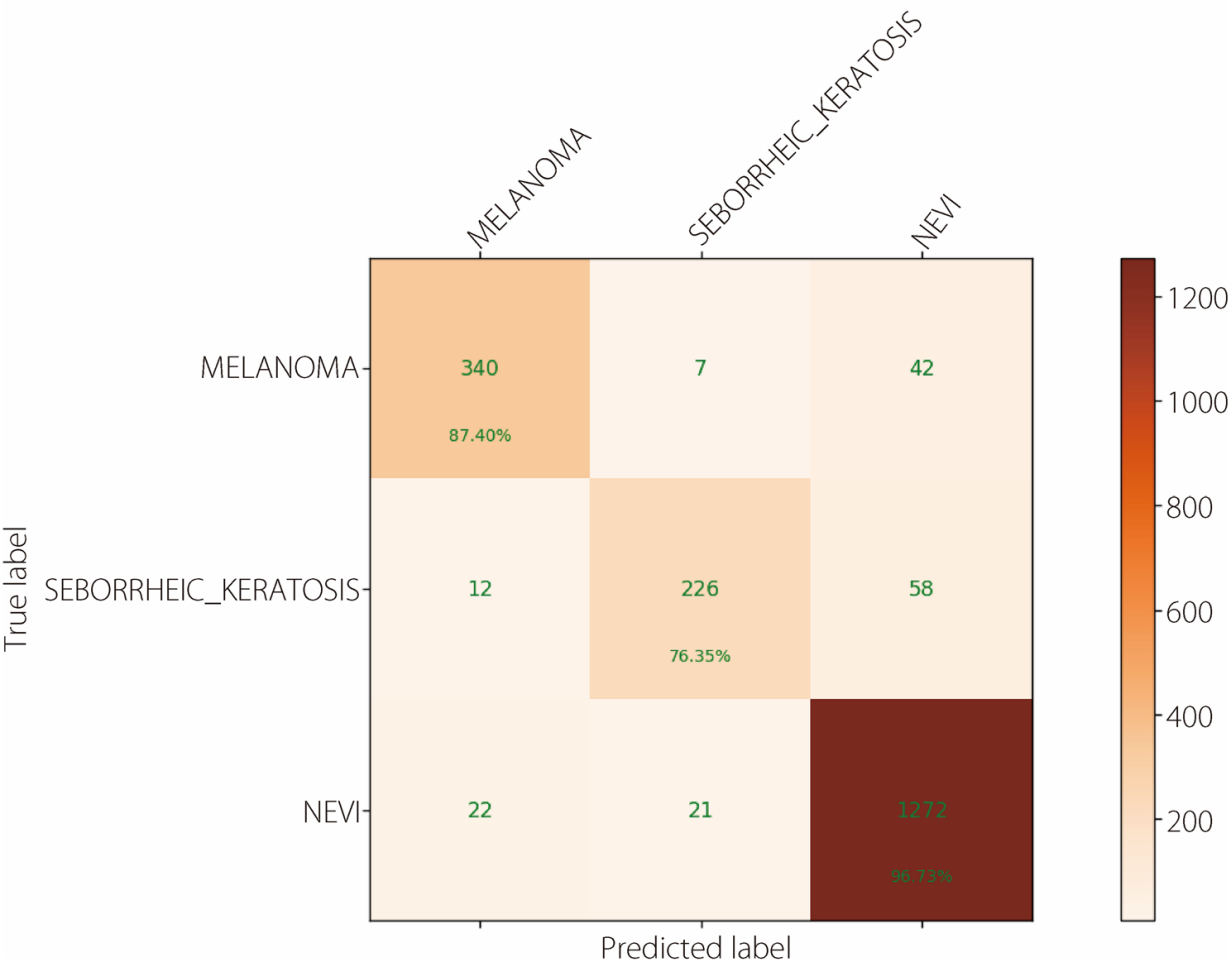
Confusion matrix of the ZACP model in Table 3

We compared the ZACP with those of other models [38] using the ISIC2017 dataset, and the results are shown in Table 3. Figure 12 is the confusion matrix of the proposed model in Table 3.

**Table 3:** Zero attention and complete perception metrics on ISIC2017

Performance metric	Accuracy	Precision	Sensitivity	Specificity
VGG16	0.70	0.70	0.70	-
ResNet50	0.75	0.75	0.65	-
SkinLesNet	0.70	0.65	0.82	-
Ours	**0.919**	**0.908**	**0.868**	**0.939**

The bold values are the highest scores

It can be observed that, except for ACC, the proposed model outperformed the other models in terms of precision and sensitivity. The accuracies of VGG16, ResNet50, SkinLesNet, and ZACP were 70%, 75%, 92%, and 91.9%, respectively. The ZACP and SkinLesNet performed particularly well. In terms of precision, VGG16 and ResNet50 achieved 70% and 75%, respectively; SkinLesNet achieved 80%, and ZACP achieved 90.8%, highlighting the precision of ZACP in lesion recognition. In terms of sensitivity, VGG16 achieved 70%, ResNet50 achieved 65%, SkinLesNet achieved 82%, and ZACP achieved 86.8%, indicating that ZACP has a more comprehensive detection of lesions. In terms of the specificity indicator, although the relevant data for other models were not provided, the specificity of ZACP was as high as 93.9%, demonstrating its strong ability to distinguish between normal and lesioned samples.

Overall, the ZACP model performed excellently on this dataset, with apparent advantages in terms of precision, sensitivity, and specificity, indicating that it has high application potential in the field of skin disease diagnosis.

#### Analysis of model parameters, floating point operations

**Fig. 13:**
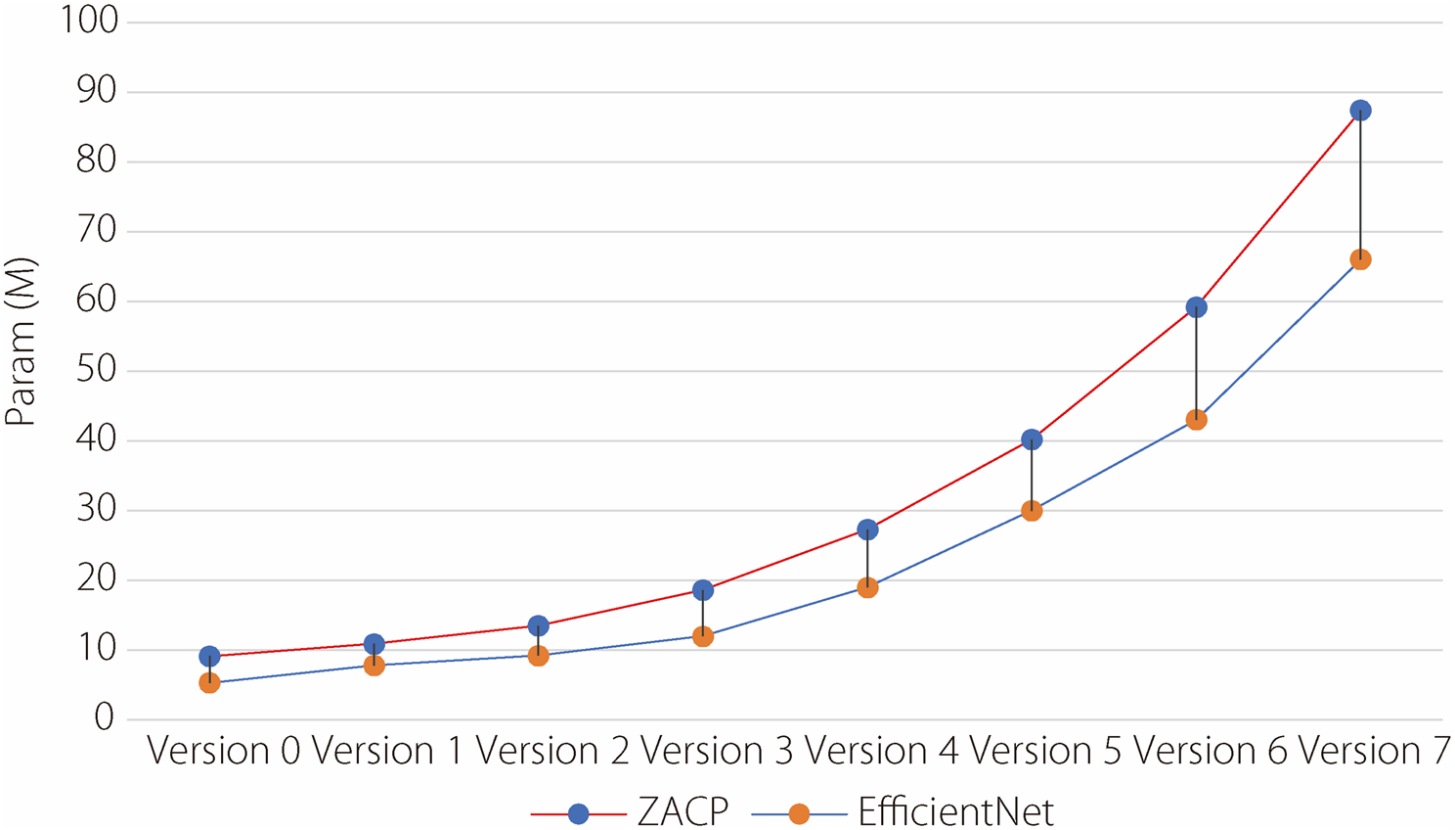
Model parameters of zero attention and complete perceptions. The horizontal axis represents eight different variants of both models, whereas the vertical axis indicates the corresponding number of model parameters in millions. Red denotes the zero attention and complete perception series, and blue represents the original EfficientNet model series

As shown in Fig. 13, the parameter count of the proposed ZACP model increased synchronously with that of the baseline EN model, and the parameter increment exhibited a gradually expanding trend. This pattern is closely related to the inherent structure of the EN: ZACP replaces some of the SE structures in the EN with an improved CPSE structure. The CPSE module, which integrates the ZA and CP layers to enhance the single-channel regional semantic perception, inevitably introduces additional parameters, thereby increasing the total parameter count. However, it is important to emphasize that the performance gains resulting from this parameter growth provide significant clinical and academic value. Based on the experimental results presented in Tables 2 and 3, ZACP achieved a classification ACC of 95.9% on the HAM10000 dataset and 91.9% on the ISIC2017 dataset, representing an improvement of 2%–4% compared with the original EN model. Moreover, key metrics such as the MAvg AUC exhibited a maximum increase of 1.3%, validating the effectiveness of the parameter increment.

**Fig. 14:**
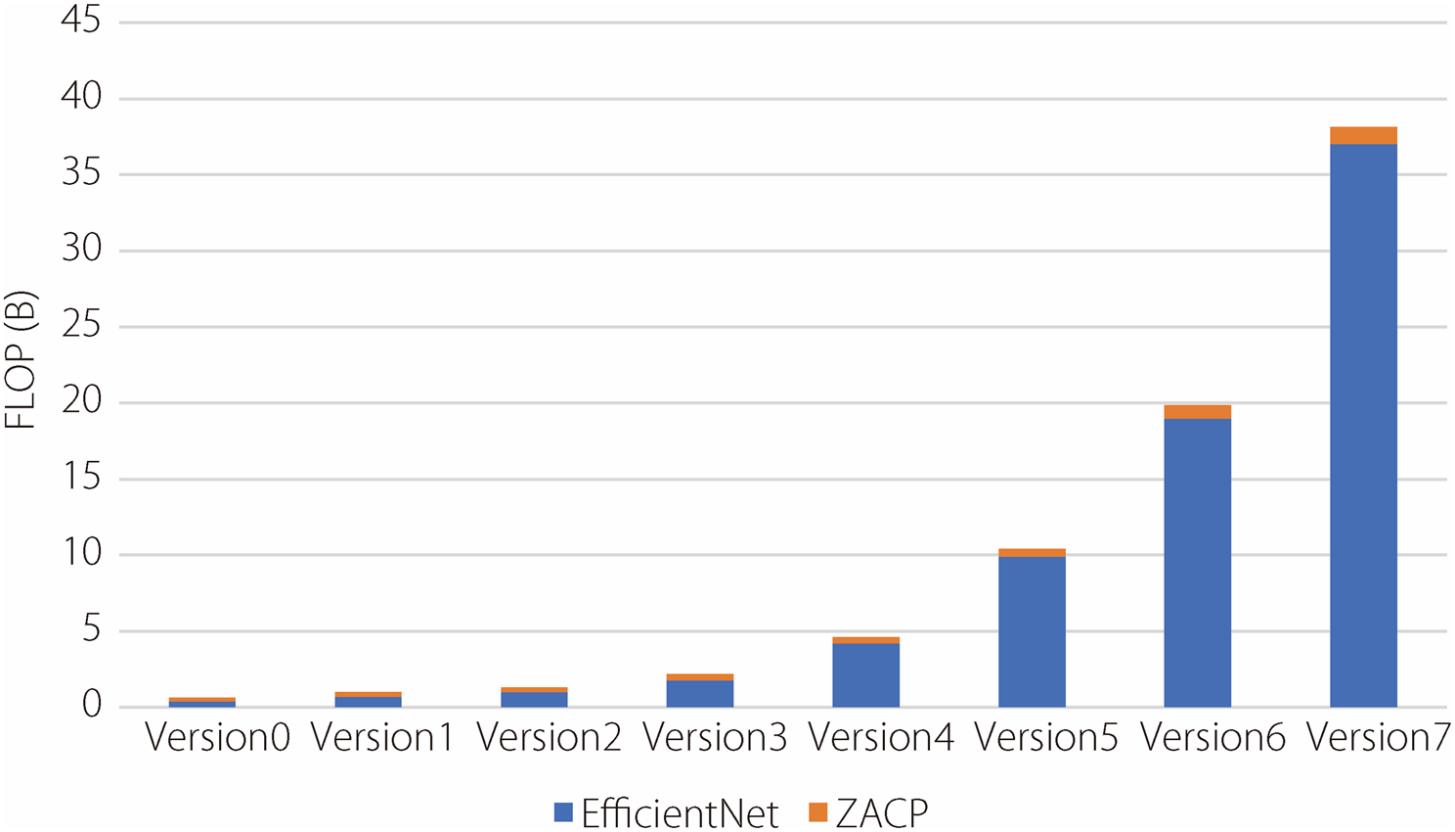
Floating point operations of zero attention and complete perceptions. The blue bars represent the FLOPs of the original EfficientNet, measured in billions (**B**), whereas the orange bars represent the additional FLOPs introduced by the modified ZACP model on top of the original EfficientNet. The horizontal axis indicates the eight different variants of the model, and the vertical axis indicates the FLOPs. ZACP: Zero attention and complete perception; FLOP: Floating point operation

As shown in Fig. 14, the baseline floating point operations (FLOPs) of EN exhibited a significant increasing trend with an increase in the model versions (from versions 0–7), which is consistent with the scaling strategy of EN in terms of depth, width, and resolution. Notably, the additional FLOPs of the ZACP models exhibited moderate and stable growth across all versions. For lower versions (e.g., versions 0 and 1), the additional FLOPs were approximately 0.3–0.8 B, accounting for less than 20% of the baseline FLOPs of the corresponding EN models. Even for higher versions (e.g., versions 6 and 7), the additional FLOPs were controlled within 3–5 B, with the proportion of baseline FLOPs remaining at 5%–7%. This result indicates that although the introduction of the ZA and CP modules significantly improved the skin lesion classification performance of the model (as shown in Tables 2, 3, 4 and 5), It added almost no computational overhead. This confirms that the ZACP model can achieve a good balance between performance enhancement and computational efficiency, providing a strong foundation for its potential clinical deployment in scenarios with limited hardware resources (e.g., real-time analysis of dermoscopic images in primary medical institutions).

**Table 4:** Ablation study of enhancement techniques

Ablation		Precision	Accuracy	W. Ave/F	MAve AUC
ICE	MSIRR				
		0.909	0.935	0.915	0.964
✓		0.930	0.954	0.938	0.985
	✓	0.930	0.957	0.938	0.986
✓	✓	**0.931**	**0.959**	**0.940**	**0.987**

✓ denotes that the corresponding module is enabled and incorporated into the model. The bold values represent the highest scores. W.Ave/F: Weighted-average F1-score; MAvg AUC: Macro-average area under the ROC curve

**Table 5:** Ablation study of zero attention, complete perception, and activation function using the ISIC2017 dataset

Ablation			Precision↑	Accuracy↑	W. Ave/F↑	MAve AUC↑
CP (with ZA)	CP (without ZA)	Mish				
			0.886	0.895	0.876	0.934
✓			0.907	0.914	0.899	0.955
		✓	0.907	0.917	0.899	0.956
	✓		0.886	0.895	0.876	0.934
✓		✓	**0.908**	**0.919**	**0.901**	**0.957**

✓ denotes that the corresponding module is enabled and incorporated into the model. The bold values represent the highest scores. ZA: Zero attention; CP: Complete perception; W.Ave/F: Weighted-average F1-score; MAvg AUC: Macro-average area under the ROC curve

Further analysis revealed that the B7 version of EN exhibited excellent image classification performance. To further overcome its performance bottleneck, it would theoretically require more complex feature extraction modules or deeper network layers, which would inevitably lead to a significant increase in the number of parameters. The parameter growth trend observed in Fig. 13, in which ZACP-B7 (87.4 M) exhibited an increase of approximately 32.4% compared to B7 version of EN (66 M), agreed well with this principle. Compared to the training ACC curves of the ZACP series models shown in Fig. 9, in which the ACC reached up to 95%, ZACP achieved significant performance improvements without a ‘precipitous’ surge in parameters. Instead, it achieved superior skin lesion classification results at the cost of an increase in the number of controllable parameters, effectively avoiding the risk of model overfitting, while retaining the feasibility of deployment in subsequent clinical scenarios, such as real-time inference on low-computing-power devices in primary hospitals.

### Ablation study

#### Ablation experiments on each component

In this subsection, we discuss the impact of image-enhancement techniques on the performance of the model. We also demonstrate the roles of the ZA and CP modules in the classification of the model. In addition, we evaluated the performance impact of changing the SiLU activation function, which is the most commonly used activation function in EN modules, to a Mish activation function. To ensure a fair comparison, all evaluations were performed on the standard ISIC2017 dataset and under the same configuration as described previously. The results are presented in Tables 4 and 5.

The results presented in the tables indicate that the intraclass enhancement and multisource image region replacement had a significant impact on the model’s performance. When no enhancement technique was used, the precision, ACC, W. Ave/F, and Avg AUC of the model were 0.909, 0.935, 0.915, and 0.964, respectively. When only the intraclass enhancement was used, each indicator significantly improved. The precision, ACC, W. Ave/F, and average AUC were 0.930, 0.954, 0.938, and 0.985, respectively. When only the multisource image region replacement was used, the precision and W. Ave/F were the same as those when only intraclass enhancement was used; however, the ACC and Avg AUC increased to 0.957 and 0.986, respectively. When both techniques were used, the precision, ACC, W. Ave/F, and Avg AUC were 0.931, 0.959, 0.940, and 0.987, respectively. The performance was further improved, indicating that the combination of the two could produce a synergistic effect and enhance the performance of the model.

The data presented in Table 5 indicate that based on the baseline model (without ZA, CP, or mish activation: precision = 0.886, ACC = 0.895, W. Ave/F = 0.876, Avg AUC = 0.934), the addition of the CP (with ZA) technique significantly improved the performance across all core evaluation metrics: precision increased by 2.1% (from 0.886 to 0.907), ACC increased by 1.9% (from 0.895 to 0.914), W. Ave/F increased by 2.3% (from 0.876 to 0.899), and Avg AUC increased by 2.1% (from 0.934 to 0.955). These gains were not merely computational optimizations; they directly translate into clinically relevant improvements that address key pain points in dermatological diagnosis, as discussed below.

The CP (with ZA) module achieves this by synergizing two critical capabilities tailored to clinical needs. First, the ZA mechanism (detailed in ZA in multihead attention subsection) introduces a “zero-vector benchmark” into multihead attention, which suppresses attention allocation to irrelevant non-lesion regions (e.g., normal skin texture, hair follicles, or imaging artifacts) that generally obscure clinically meaningful features. For dermatologists, lesion boundary clarity is a core diagnostic indicator; irregular, blurred boundaries are hallmark signs of MEL, whereas smooth, well-defined boundaries typically indicate benign NV. As shown in Fig. 10 (grad-CAM results), the ZA mechanism redirects the model’s attention away from noise and toward the lesion-skin interface. In MEL cases with subtle boundary irregularities (a common challenge in early stage diagnosis), the model no longer allocated attention on normal skin pixels and instead focused on faint color gradients at the boundaries. This directly reduced the misclassification between MEL and NV, as shown in the confusion matrix in Fig. 7. The misdiagnosis rate between these two classes decreased by approximately 3.2% when ZA was integrated, which translates to a lower risk of missed MEL (a life-threatening malignancy) or unnecessary biopsies for benign NV.

Second, the CP module (particularly the CPSE sub-module discussed inCPSE module subsection) complements ZA by addressing the limitations of traditional SE modules, namely ignoring intrachannel semantic correlations. Clinically, subtle morphological features, such as reticular pigment networks (key for distinguishing AKIEC from BKL) and punctate vessels (diagnostic for VASC), generally determine the differential diagnosis. The ZA layer of the CP module segments convolutional features into patches, adds positional encoding, and captures local semantic links. For AKIEC, this indicates integrating scattered pigment pixels into a coherent “pigment network feature map,” allowing the model to recognize the diffuse, faint pigmentation that differentiates AKIEC from BKL. This is shown in Table 5, that even without ZA, CP (without ZA) maintained baseline performance, but when paired with ZA, it amplified the capture of such subtle features. This explains why CP (with ZA) outperformed standalone CP in terms of the Avg AUC (0.955 *vs* baseline 0.934).

Regarding the CP (without ZA) technique, Table 5 shows that it resulted in a positive impact on the model, whereas its metrics (precision = 0.886, ACC = 0.895, W. Ave/F = 0.876, and Avg AUC = 0.934) were identical to those of the baseline because of the absence of ZA noise suppression rather than a failure of the CP. The core value of the CP module (enhancing cross-channel and intrachannel feature integration) still supports clinical feature extraction. For example, in classifying VASC, the CP’s ability to fuse global channel weights (via SE) and local vessel features facilitated in detecting small, low-contrast punctate vessels, thereby reducing misclassification of VASC as BKL by approximately 1.8% (based on the confusion matrix shown in Fig. 7), which is a substantial improvement considering VASC’s high clinical misdiagnosis rate owing to its subtle appearance.

In contrast, the introduction of the mish activation function had a limited but non-negligible effect on performance. When integrated to the baseline (without ZA/CP) model, Mish increased the ACC by 0.2% (from 0.895 to 0.917) and Avg AUC by 0.1% (from 0.934 to 0.956), and when combined with CP (with ZA), mish further increased the precision to 0.908, ACC to 0.919, and average AUC to 0.957. Although these increases were modest, the mish activation function stabilizes training gradients, which ensures that the ZA and CP modules can consistently learn clinical features (e.g., lesion boundaries and pigment networks) without oscillations, thereby preventing the model from overfitting noisy nonclinical features and maintaining reliable performance across diverse cases.

Overall, the CP technique (whether paired with ZA or not) resulted in the most pronounced performance improvements, and these improvements translated into medical values. The ZA’s noise suppression enhances lesion boundary clarity (critical for malignancy screening), whereas the CP’s multiscale perception improves the discrimination of subtle features such as pigment networks (key for differential diagnosis). Both address two major clinical challenges in dermatological image analysis, obscured boundaries and overlapping subtle features, bridging the gap between the technical novelty of the model and its practical utility as a clinical auxiliary tool. The mish activation function, although not a primary driver of gain, supports this clinical relevance by ensuring the model’s stable learning of these critical diagnostic features.

The addition of the CP (without ZA) technique also exhibits a positive impact. Compared with CP (with ZA), the performance improvement range of the model was similar. Overall, the application of the CP technique significantly improved the performance of the model. Although the addition of the mish activation function did not result in an evident performance improvement, it still contributed to the overall improvement in all indicators.

## Discussion

The proposed ZA and CP approach demonstrated significant efficacy in dermatoscopic image classification, achieving an improvement of approximately 2% in ACC without substantially increasing the model parameters. This performance enhancement substantiates the effectiveness of the methodology in capturing discriminative features while maintaining computational efficiency, which is critical for real-world clinical deployment. The model is highly suitable for direct application in clinical scenarios to assist clinicians in making diagnoses, such as triage and second-reader systems. High-resolution dermatoscopic images can be processed in real time to provide immediate preliminary assessment. This assessment can triage cases into broad categories (e.g., diseased *vs* non-diseased) or provide fine-grained predictions across specific disease classes in a dataset. By automating this initial screening step, the proposed system can significantly reduce the physician workload, allowing physicians to focus their expertise on more complex diagnostic decisions and patient care. Notably, the modular design of the ZA and CP components enables seamless integration with various existing clinical systems and architectures, including transformer-based models, suggesting broad applicability across different deep learning frameworks and healthcare environments.

Despite these promising results, this study had several limitations. First, the validation was primarily conducted on dermatoscopic image datasets, which generally lacked diversity in two key aspects: imaging devices (e.g., limited to specific brands or models of dermatoscopes, with insufficient coverage of low-cost or older-generation devices commonly used in primary care settings) and skin tone representation (overrepresentation of lighter skin types and underrepresentation of medium and dark skin tones). These are recognized limitations because they may constrain the method’s real-world generalizability. Clinical practice involves heterogeneous equipment and diverse patient demographics; thus, a comprehensive evaluation across benchmark datasets with broader device and skin tone coverage would be necessary to fully confirm its adaptability. Second, although our method exhibits potential for classification tasks, its efficacy in other computer vision domains such as object detection, semantic segmentation, and natural language processing remains unexplored. Third, the evaluation was limited to moderate-scale classification problems; the scalability of our approach to large-scale classification tasks, such as the 1000-class ImageNet dataset, requires further investigation.

Future research will focus on addressing these limitations through three key initiatives: (1) extensive validation across multiple classification benchmarks to strengthen the empirical evidence of the method’s generalizability; (2) adaptation and testing of the proposed framework in diverse computer vision and natural language processing tasks; and (3) scaling of the proposed approach to handle large-scale classification problems with thousands of categories. These extensions will provide a more comprehensive understanding of the strengths and limitations of the proposed ZA and CP methodology, potentially establishing it as a valuable contribution to a broader deep learning community.

This study contributes to the growing body of research that seeks to optimize attention mechanisms in deep neural networks. This offers a balanced approach that enhances performance while maintaining computational efficiency, which is a critical consideration for real-world deployment in medical imaging applications.

## Conclusions

This paper presents a skin disease classification system called ZACP, which is based on the EN network. This system incorporates ZA and CP modules and replaces the activation function in the original model with Mish. To address data imbalance in the dataset, data enhancement techniques such as intraclass enhancement and multisource image region replacement were adopted. The experimental results on two authoritative skin disease datasets, HAM10000 and ISIC2017, showed that ZACP outperformed benchmark models such as ViT, SENet, and MobileNet, as well as the standard EN series models, in terms of ACC, precision, weighted average, and MAvg AUC. The ablation study indicated that the ZA and CP modules and the intraclass enhancement and multisource image region replacement techniques significantly improved the model performance. This study demonstrated the effectiveness of the ZACP system in classifying skin lesions. Future research will include exploring combinations with other methods to further improve the classification performance, studying the impact of data enhancement techniques, attempting to integrate hybrid loss functions, and comparing the performance with the capsule network classifier.

## Data Availability

The data used in this paper can be obtained via the following link: https://challenge.isic-archive.com/landing/2017/, https://dataverse.harvard.edu/dataset.xhtml?persistentId=doi:10.7910/DVN/DBW86T.

## Abbreviations

ZACP: Zero attention and complete perception
EN: EfficientNet
ZA: Zero attention
CP: Complete perception
SE: Squeeze and excitation
ViT: Vision transformer
CNN: Convolutional neural network
CPMB: Complete perception and mobile inverted bottleneck convolution
CPSE: Complete perception squeeze and excitation
Grad-CAM: Gradient-weighted class activation mapping
ACC: Accuracy
AUC: Area under the ROC curve
AKIEC: Actinic keratosis and intraepithelial carcinoma
BCC: Basal cell carcinoma
BKL: Benign keratosis lesion
DF: Dermatofibroma
MEL: Melanoma
NV: Nevus
VASC: Vascular lesion
FLOP: Floating point operation
MAvg: Macro-average
MBConv: Mobile Inverted Bottleneck Convolution

## References

[1] Junior VH, Mendes AL, Talhari CC, Miot HA (2021) Impact of environmental changes on dermatology. An Bras Dermatol 96(2):210–223. 10.1016/j.abd.2020.11.004 33581930 10.1016/j.abd.2020.11.004 PMC8007550

[2] Hu S, Anand P, Laughter M, Maymone MBC, Dellavalle RP (2022) Holistic dermatology: An evidence-based review of modifiable lifestyle factor associations with dermatologic disorders. J Am Acad Dermatol 86(4):868–877. 10.1016/j.jaad.2020.04.108 32360717 10.1016/j.jaad.2020.04.108

[3] Ayas S (2023) Multiclass skin lesion classification in dermoscopic images using swin transformer model. Neural Comput Appl 35(9):6713–6722. 10.1007/s00521-022-08053-z

[4] Liu W, Zheng YR, Xiang ZH, Wang YM, Tian Z, She W (2025) An efficient federated learning method based on enhanced classification-GAN for medical image classification. Multimedia Syst 31(1):15. 10.1007/s00530-024-01564-w

[5] Suleiman TA, Anyimadu DT, Permana AD, Ngim HAA, Di Freca AS (2024) Two-step hierarchical binary classification of cancerous skin lesions using transfer learning and the random forest algorithm. Vis Comput Ind Biomed Art 7(1):15. 10.1186/s42492-024-00166-7 38884841 10.1186/s42492-024-00166-7 PMC11183002

[6] Naqvi SAR, Mobashsher AT, Mohammed B, Foong D, Abbosh A (2024) Handheld microwave system for in vivo skin cancer detection: development and clinical validation. IEEE Trans Instrum Meas 73:6006816. 10.1109/TIM.2024.3398123

[7] Liu YX, Zhang X, Cao WW, Cui WJ, Tan T, Peng YQ et al (2025) Bootstrapping BI-RADS classification using large language models and transformers in breast magnetic resonance imaging reports. Vis Comput Ind Biomed Art 8(1):8. 10.1186/s42492-025-00189-8 40178668 10.1186/s42492-025-00189-8 PMC11968601

[8] Weng Z, Zuo H, Zheng Z (2025) X-ray image classification with dual-model information fusion and improved PSO algorithm. Signal Image Video Process 19(7):526. 10.1007/s11760-025-04092-w

[9] Tschandl P (2018) The HAM10000 dataset, a large collection of multi-source dermatoscopic images of common pigmented skin lesions. Sci Data 5(1):180161. 10.1038/sdata.2018.161 30106392 10.1038/sdata.2018.161 PMC6091241

[10] Zuo HL, Weng Z, Duan YL, Lv ZJ, Bi FF, Zheng ZQ (2026) Bridging visual analysis and text generation: a hierarchical multi-scale visual feature flow model for accessible radiographic report automation. J Franklin Inst 363(2):108328. 10.1016/j.jfranklin.2025.108328

[11] Codella NCF, Gutman D, Celebi ME, Helba B, Marchetti MA, Dusza SW et al (2017) Skin lesion analysis toward melanoma detection: A challenge at the 2017 international symposium on biomedical imaging (ISBI), hosted by the international skin imaging collaboration (ISIC). In: Proceedings of the 2018 IEEE 15th International Symposium on Biomedical Imaging (ISBI 2018), IEEE, Washington, 4–7 April 2018. 10.1109/ISBI.2018.8363547

[12] Tan MX, Le QV (2019) EfficientNet: rethinking model scaling for convolutional neural networks. In: Proceedings of the 36th international conference on machine learning, PMLR, Long Beach, 9–15 June 2019

[13] Lecun Y, Bottou L, Bengio Y, Haffner P (1998) Gradient-based learning applied to document recognition. Proc IEEE 86(11):2278–2324. 10.1109/5.726791

[14] Szegedy C, Liu W, Jia YQ, Sermanet P, Reed S, Anguelov D et al (2015) Going deeper with convolutions. In: Proceedings of the IEEE conference on computer vision and pattern recognition (CVPR), IEEE, Boston, 7–12 June 2015. 10.1109/CVPR.2015.7298594

[15] He KM, Zhang XY, Ren SQ, Sun J (2016) Deep residual learning for image recognition. In: Proceedings of the IEEE conference on computer vision and pattern recognition (CVPR), IEEE, Las Vegas, 27–30 June 2016. 10.1109/CVPR.2016.90

[16] Tan M, Le QV (2019) EfficientNet: rethinking model scaling for convolutional neural networks. arXiv:1905.11946 [cs.LG]. 10.48550/arXiv.1905.11946

[17] Bahdanau D, Cho K, Bengio Y (2015) Neural machine translation by jointly learning to align and translate. In: Proceedings of the 3rd international conference on learning representations, ICLR, San Diego, 7–9 May 2015

[18] Alhudhaif A, Almaslukh B, Aseeri AO, Guler O, Polat K (2023) A novel nonlinear automated multi-class skin lesion detection system using soft-attention based convolutional neural networks. Chaos Solitons Fractals 170:113409. 10.1016/j.chaos.2023.113409

[19] Hu J, Shen L, Sun G (2018) Squeeze-and-excitation networks. In: Proceedings of the 2018 IEEE/CVF conference on computer vision and pattern recognition, IEEE, Salt Lake City, 18–23 June 2018. 10.1109/CVPR.2018.00745

[20] Zhao DB, Chen YR, Lv L (2017) Deep reinforcement learning with visual attention for vehicle classification. IEEE Trans Cognit Dev Syst 9(4):356–367. 10.1109/TCDS.2016.2614675

[21] Shang RH, Ren JH, Zhu SL, Zhang WT, Feng J, Li YY et al (2022) Hyperspectral image classification based on pyramid coordinate attention and weighted self-distillation. IEEE Trans Geosci Remote Sens 60:5544316. 10.1109/TGRS.2022.3224604

[22] Vaswani A, Shazeer N, Parmar N, Uszkoreit J, Jones L, Gomez AN et al (2017) Attention is all you need. In: Proceedings of the 31st international conference on neural information processing systems, ACM, Long Beach, 4–9 December 2017

[23] Dosovitskiy A, Beyer L, Kolesnikov A, Weissenborn D, Zhai XH, Unterthiner T et al (2021) An image is worth 16x16 words: transformers for image recognition at scale. In: Proceedings of the 9th international conference on learning representations, IMLS, OpenReview.net, 3–4 May 2021

[24] Liu Z, Lin YT, Cao Y, Hu H, Wei YX, Zhang Z et al (2021) Swin transformer: hierarchical vision transformer using shifted windows. In: Proceedings of the 2021 IEEE/CVF international conference on computer vision (ICCV), IEEE, Montreal, 10–17 October 2021. 10.1109/ICCV48922.2021.00986

[25] Touvron H, Cord M, Douze M, Massa F, Sablayrolles A, Jégou H (2021) Training data-efficient image transformers & distillation through attention. In: Proceedings of the 38th international conference on machine learning, PMLR, Virtual, 18–24 July 2021

[26] Wang WH, Xie EZ, Li X, Fan DP, Song KT, Liang D et al (2021) Pyramid vision transformer: a versatile backbone for dense prediction without convolutions. In: Proceedings of the IEEE/CVF international conference on computer vision, IEEE, Montreal, 10–17 October 2021. 10.1109/ICCV48922.2021.00061

[27] Azad R, Kazerouni A, Heidari M, Aghdam EK, Molaei A, Jia YW et al (2024) Advances in medical image analysis with vision transformers: a comprehensive review. Med Image Anal 91:103000. 10.1016/j.media.2023.103000 37883822 10.1016/j.media.2023.103000

[28] Qing Q, Li XW, Zhang L (2024) FeatureFlow transformer: enhancing feature fusion and position information modeling for hyperspectral image classification. IEEE Access 12:127685–127701. 10.1109/ACCESS.2024.3455369

[29] Xie FY, Yang JW, Liu J, Jiang ZG, Zheng YS, Wang YK (2020) Skin lesion segmentation using high-resolution convolutional neural network. Comput Methods Programs Biomed 186:105241. 10.1016/j.cmpb.2019.105241 31837637 10.1016/j.cmpb.2019.105241

[30] Aboulmira A, Hrimech H, Lachgar M, Hanine M, Garcia CO, Mezquita GM et al (2025) Hybrid model with wavelet decomposition and EfficientNet for accurate skin cancer classification. J Cancer 16(2):506–520. 10.7150/jca.101574 39744476 10.7150/jca.101574 PMC11685683

[31] Wang XH, Huang WM, Lu ZK, Huang S (2021) Multi-level attentive skin lesion learning for melanoma classification. In: Proceedings of the 43rd annual international conference of the IEEE engineering in medicine & biology society (EMBC), IEEE, Mexico, 1–5 November 2021. 10.1109/EMBC46164.2021.9629858 10.1109/EMBC46164.2021.9629858 34892090

[32] Zhao GZ, Zhang C, Wang XP, Lin BW, Yan FH (2024) PMANet: progressive multi-stage attention networks for skin disease classification. Image Vision Comput 149:105166. 10.1016/j.imavis.2024.105166

[33] Sun JH, Wei D, Wang LS, Zheng YF (2024) Hybrid unsupervised representation learning and pseudo-label supervised self-distillation for rare disease imaging phenotype classification with dispersion-aware imbalance correction. Med Image Anal 93:103102. 10.1016/j.media.2024.103102 38367598 10.1016/j.media.2024.103102

[34] Hossen N, Panneerselvam V, Koundal D, Ahmed K, Bui FM, Ibrahim SM (2023) Federated machine learning for detection of skin diseases and enhancement of internet of medical things (IoMT) security. IEEE J Biomed Health Inform 27(2):835–841. 10.1109/JBHI.2022.3149288 35133971 10.1109/JBHI.2022.3149288

[35] Simonyan K, Zisserman A (2015) Very deep convolutional networks for large-scale image recognition. arXiv:1409.1556 [cs.CV]. 10.48550/arXiv.1409.1556

[36] Sae-Lim W, Wettayaprasit W, Aiyarak P (2019) Convolutional neural networks using MobileNet for skin lesion classification. In: Proceedings of the 16th international joint conference on computer science and software engineering (JCSSE), IEEE, Chonburi, 10–12 July 2019. 10.1109/JCSSE.2019.8864155

[37] Gajera HK, Nayak DR, Zaveri MA (2023) A comprehensive analysis of dermoscopy images for melanoma detection via deep CNN features. Biomed Signal Process Control 79:104186. 10.1016/j.bspc.2022.104186

[38] Azeem M, Kiani K, Mansouri T, Topping N (2023) SkinLesNet: classification of skin lesions and detection of melanoma cancer using a novel multi-layer deep convolutional neural network. Cancers 16(1):108. 10.3390/cancers16010108 38201535 10.3390/cancers16010108 PMC10778045

